# Haplotype-resolved genome reveals haplotypic variation and the biosynthesis of medicinal ingredients in *Areca catechu* L.

**DOI:** 10.1186/s43897-025-00146-2

**Published:** 2025-05-02

**Authors:** Chao Wang, Lei Tan, Zhonghui Zhang, Xianggui Li, Linghao Xia, Peng Cao, Haiyang Tong, Xumin Ou, Shixuan Li, Jianing Zhang, Chun Li, Jun Yang, Wen-Biao Jiao, Shouchuang Wang

**Affiliations:** 1 National Key Laboratory for Tropical Crop Breeding, School of Breeding and Multiplication (Sanya Institute of Breeding and Multiplication), Hainan University, Sanya Hainan, 572025 China; 2 National Key Laboratory for Tropical Crop Breeding, College of Tropical Agriculture and Forestry, Hainan University, Sanya Hainan, 572025 China; 3 National Key Laboratory for Germplasm Innovation & Utilization of Horticultural Crops, Huazhong Agricultural University, Wuhan, 430070 China; 4 Hubei Hongshan Laboratory, Wuhan, 430070 China

**Keywords:** *Areca catechu*, Haplotype-resolved genome, Ancestral karyotype, Pyridine alkaloid, Flavonoid, *N*-methyltransferase

## Abstract

*Areca catechu,* as a traditional Chinese medicine, contains a high concentration of therapeutic compounds. However, the biosynthesis of these compounds is largely unexplored. We present a haplotype-resolved genome assembly and annotation for *A. catechu*, with chromosome-level genome sizes of 2.45 Gb (*Ac. Hap1*) and 2.49 Gb (*Ac. Hap2*). A comparative analysis of the haplotypes revealed significant divergence, including multiple Mb-level large inversions. Furthermore, *A. catechu* shared two whole genome duplications with other palm plants and its genome size had increased due to the insertion of transposons within the last 2.5 million years. By integrating transcriptomics and metabolomics, two tandem genes (*AcGNMT1* and *AcGNMT2*) were negatively associated with guvacine and trigonelline in gene-metabolite interaction network. *AcGNMT1*, *AcGNMT2* and their three homologous genes were involved in the conversion of guvacine to arecoline. Further analyses tested the function of *AcUGT71CE15*, *AcUGT74CJ38*, *AcUGT87EE5* and *AcUGT83S982* as glucosyltransferases, and *AcUGT78AP14* was identified as a rhamnosyltransferase involved in flavonol glycosylation. Our study provides a high-quality genome of *A. catechu*, characterizes the arecoline biosynthetic pathway and expands the understanding of the diversity of UDP-glucosyltransferase and UDP-rhamnosyltransferase, offering insights into the potential of *A. catechu* for the biosynthesis of bioactive compounds.

**Supplementary Information:**

The online version contains supplementary material available at 10.1186/s43897-025-00146-2.

## Core

Two haplotype-resolved genomes reported in this study provide insights into genetic differences between homologous chromosomes. Based on metabolomics and transcriptomics, a gene-metabolite network was constructed and provided a resource for the analysis of key metabolic pathways in *A. catechu*. This work lays a foundation for further research and the utilization of important tropical medicinal resources.

## Gene and accession number

The whole genome sequence data reported in this paper have been deposited in the Genome Warehouse in National Genomics Data Center under BioProject accession number PRJCA033974. The RNA-seq presented in this study are available in NCBI under the BioProject accession PRJNA929020. The raw metabolomics data are available via http://www.ebi.ac.uk/metabolights/MTBLS6967.

## Introduction

The Arecaceae family, also known as Palmaceae, contains a wide range of plants recognized for their economic, ornamental, and medicinal value, such as *Trachycarpus nana*, *Cocos nucifera*, and *Elaeis guineensis*. *Areca catechu* (2*n* = 32) belongs to the Arecacae family and is mainly distributed in tropical regions, including eastern Africa, southern and southeastern Asia, and some regions in the Pacific Ocean (Peng et al. 2015; Yang et al. 2021b). In 2022, *A. catechu* occupied an area of approximately 1.21 million hectares globally, with a total yield of 2.54 million tons (https://www.fao.org/faostat/en/ Accessed February 2024). Areca nuts are used for chewing and traditional herbal medicine, practices that date back to the Han Dynasty of China, and *A. catechu* is distinguished from other palm plants by its high content of arecoline in its nuts.

Fundamental chemical modifications of metabolites can affect the capacity of plants to adapt to environmental change and increase metabolite diversity and bioactivity in plant, which contain glycosyl, carboxyl, methyl, and hydroxyl functional groups (Jones et al. 2001; Wang et al. 2019). As a traditional Chinese medicine, areca nuts are enriched in various active ingredients such as alkaloids, flavonoids, polyphenols, and triterpenes (Yang et al. 2021a). The major bioactive compounds in areca nuts are the pyridine alkaloids (arecoline, arecaidine, guvacoline, and guvacine), which often bind with tannic acid and account for 90% of areca nuts’ total alkaloid content (Cao et al. 2020). Among them, clinical uses of arecoline include treating glaucoma, tapeworm infections, and joint inflammation and it also provides significant benefits to the nervous and cardiovascular systems (Chiu et al. 2021; Garg et al. 2014; Wang et al. 2018; Patil et al. 2002). On the basis of structural similarity, nicotinate is likely the precursor of guvacine, which is decorated by *N*-methyl or/and *O*-methyl modification to form the three other alkaloids. The biosynthesis mechanism of the pyridine alkaloids in *A. catechu* have not been fully elucidated, but this information is critical to understand arecoline biosynthesis. *A. catechu* also contains more than 100 flavonoids. Flavnoids are commonly glycosylated and, in addition to their importance to plant defenses, flavonol glycosides have various medicinal benefits (Bondonno et al. 2019; Lai et al. 2023; Liu et al. 2020). The most common flavonol aglycones include naringenin, kaempferol and quercetin, and commonly used sugar donors include UDP-glucose (UDP-Glc) and UDP-rhamnose (UDP-Rha). Although *Acat_15g017010* and *Acat_16g013670* have been identified as glucosyltransferases involved in the glycosylation of kaempferol and chrysin, few glucosyltransferases and no rhamnosyltransferases have been reported in *A. catechu* (Lai et al. 2023).

In recent years, the biosynthesis pathways of numerous important natural products in medicinal plants, such as paclitaxel biosynthesis in *Taxus*, protoberberine-type alkaloids biosynthesis in *Coptis chinensis* and Oleanane-type triterpenoids biosynthesis in *Aralia elata,* have been decoded using multi-omics analysis (genomics, transcriptomics, and metabolomics) (Xiong et al. 2021; Liu et al. 2021; Zhao et al. 2023). So far, two chromosome-level genomes for *A. catechu* have been reported, but the two assembled genomes differed dramatically (2.59 Gb vs. 2.73 Gb) because of limitations in sequencing technology (Yang et al. 2021b; Zhou et al. 2022a). Also, the two assemblies likely missed allelic variations underlying important selected traits (Zhang et al. 2021). Recently, HiFi sequencing has emerged as a method to generate haplotype-resolved genomes with more refined genetic information and long reads, which make it possible to study allelic variations (Niu et al. 2022; Hu et al. 2022). Integrating metabolomics and transcriptomics can be used to decode metabolic pathways. For example, recent work combined a characterization of metabolic changes with parallel transcriptome analysis to identify novel transcription factors during the tomato growth cycle (Luo et al. 2015; Li et al. 2020). Some research has generated and analyzed datasets that encompass transcriptomes and metabolomes (Lai et al. 2023; Zhou et al. 2023). However, information about the gene-metabolite interaction network is limited, and this information is necessary to elucidate the biosynthesis of medicinal compounds in *A. catechu*.

Here, we have assembled two haplotype-resolved genomes in *A. catechu* and identified genetic differences on homologous chromosomes. Using phylogenetic and genomic comparative analyses, we identified the evolutionary position of *A. catechu* in Monocotyledon and Arecaceae, and confirmed two whole genome duplications (WGDs) shared between species of Arecaceae. We inferred six ancient monocot karyotypes (AMKs) based on gene blocks of multi-chromosomal species and identified the main factors contributing to the specific amplification of the genome size of *A. catechu*. By integrating genomics, transcriptomics, metabolomics, and biochemical experiments, we constructed the arecoline biosynthesis pathway and identified the *AcGNMTs* involved in converting guvacine to arecoline. We also identified one rhamnosyltransferase and four glucosyltransferases of flavonoids, which contribute to the diversity of flavonol glycosides in *A. catechu*. These findings help elucidate the formation of the diverse bioactive compounds in *A. catechu* and provide a foundation for research into medical applications.

## Results

### Haplotype-resolved genome assembly and annotation of *A. catechu*

**Fig. 1:**
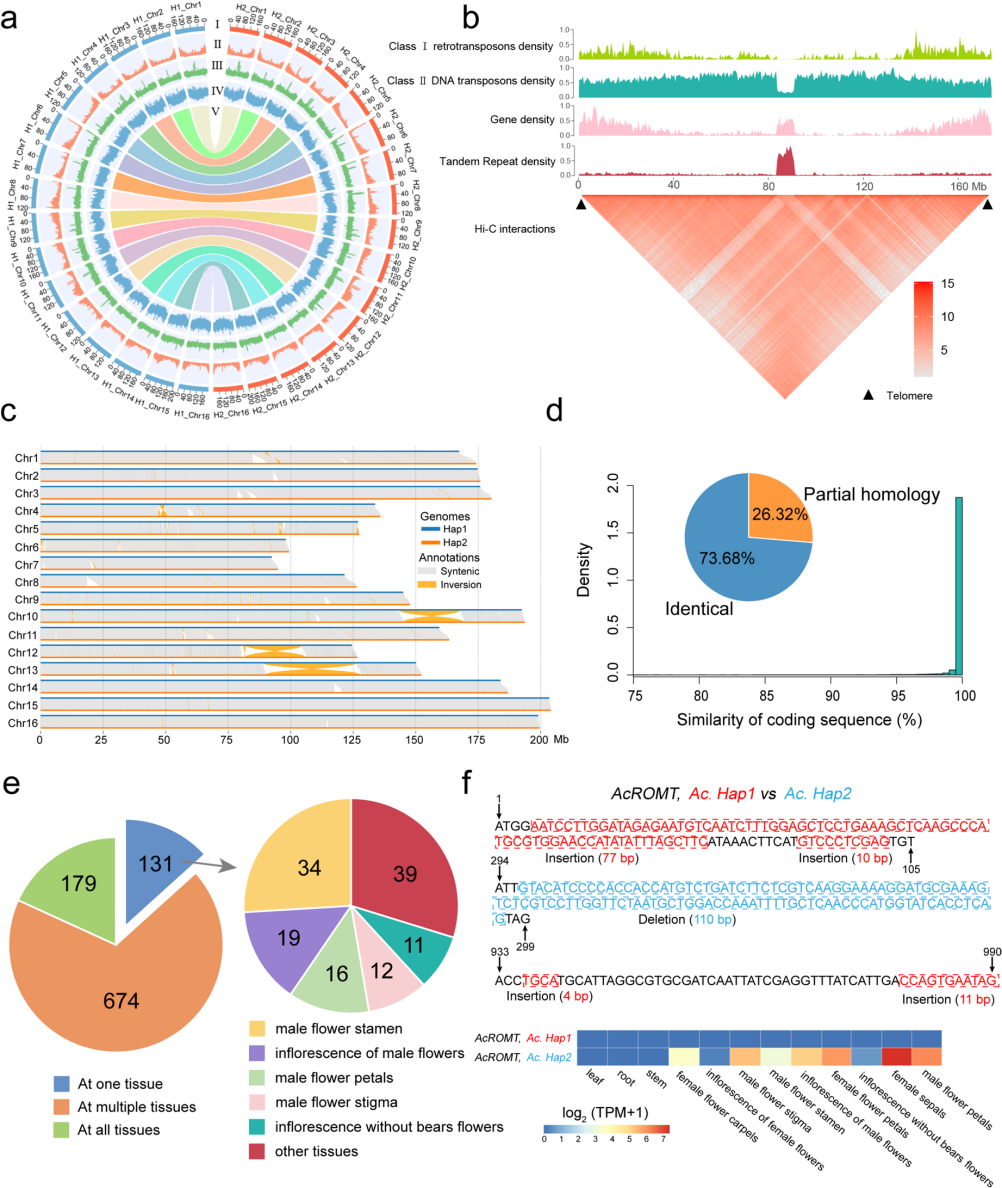
*A. catechu* genome assembly and genomic features. **a** Circos diagram of haplotype 1 (*Ac. hap1*) and haplotype 2 (*Ac. Hap2*)*.* The circles from outer to inner separately represented chromosome length (I); gene density (II); GC content (III); repeat density (IV); Colinear links (V). **b** Characterization of the putative centromere on chromosome 1 of *Ac. Hap2*. **c** The syntenic and inverted regions between two haplotypes. **d** Similatity of coding sequences for allelic gene pairs. **e** Different expression number of allelic gene pairs in twelve tissues (stem, root, leaf and different part of flowers). **f** An example of an allelic imbalance (*AcROMT*, *Hap1_ACA9G003180* vs *Hap2_ACA9G003050*) with an inconsistent expression pattern. Top, allelic variations between *Ac. hap1* and *Ac. hap2*, including 4 insertions (red, 4–77 bp) and 1 delection (blue, 110 bp). Bottom, allelic differential expression of this gene in twelve tissues

We performed PacBio HiFi and Hi-C sequencing for de novo assembly of the *A. catechu* genome. 74.99 Gb PacBio HiFi reads were obtained using the PacBio Sequel II platform, with an average length of 15,978 bp and coverage of approximately 29.4X (Table S1). The heterozygous rate of the *A. catechu* genome was 0.50%, which was slightly higher than *C. nucifera* (0.16%) and lower than previously published *A. catechu* genome (0.86%) (Wang et al. 2021; Yang et al. 2021b). Two haplotype assemblies with total lengths of 2.50 and 2.55 Gb were obtained using Hifiasm in HiFi + Hi-C mode. The two haplotypes featured contig N50 values of 41.66 and 32.00 Mb, with maximum contig lengths of 109.21 and 181.27 Mb, respectively (Table 1). Contigs of each haplotype assembly were further scaffolded with 247.66 Gb Hi-C reads, and anchored into 16 pseudochromosomes (Figure S1 and Table S2-S3) with covering rates of 97.94% and 97.59%, respectively. The final two chromosome-level haplotypes with total lengths of 2.45 Gb and 2.49 Gb were obtained (Fig. 1a). To assess the accuracy of genome assembly, the consensus quality value (QV) of the two haplotypes was calculated. The consensus QV scores of *Ac. Hap1* and *Ac. Hap2* were 65.66 and 64.71, respectively. Our results showed a higher scores than the released genome of *Areca catechu* (2.51 Gb, QV = 35.56) and *Eutrema japonicum* (1.5 Gb, QV = 51.33) (Tanaka et al. 2023; Yang et al. 2021b). Subsequently, genome quality was assessed using a series of subsequent analyses. LTR assembly index (LAI) values of the genome were 17.30 and 17.37. We also mapped RNA-seq reads to the genome with alignment rates of over 94%. In addition, benchmarking universal single-copy orthologs (BUSCO) analysis suggested that the assembly completeness values for two haplotypes were 98.2% (*Ac. Hap1*) and 98.6% (*Ac. Hap2*) (Table S4). Together, these results indicated high-quality haplotype-resolved genome assembly of *A. catechu*.

**Table 1:** Statistics of assemblies and annotations of *A. catechu* genome

	Haplotype 1 (Ac. Hap1)	Haplotype 2 (Ac. Hap2)	A. catechu V1 (2021)	A. catechu V2 (2022)
Genome size (Mb)	2,499.98	2,550.01	2,507.60	2,730.00
GC content	41.73%	41.96%	41.14%	40.80%
Contig number	657	661	7,190	-
N50 contig length (Mb)	41.66	32.00	0.87	2.78
N90 contig length (Mb)	8.71	6.60	0.17	-
Longest contig length (Mb)	109.21	181.27	7.19	-
Gene number	32,320	34,238	31,571	31,406
Average gene length (bp)	9,483.11	8,861.12	14,076.00	6,717.84
Average CDS length	216.28	217.92	220.46	233.36
Chromosomes	16	16	16	16
Exons per gene	6.60	6.47	5.42	6.75
Percent of repeat sequence	82.97%	82.98%	80.37%	69.19%
Anchored rate	97.94%	97.59%	97.90%	96.70%

Based on ab initio gene predictions, homologous protein alignments, and RNA-seq read mappings, 32,320 and 34,238 protein-coding genes were annotated (Table 1). Following comparison of homologous sequences and protein domains, approximately 94.81% and 94.58% of genes were functionally annotated (Table S5). Repetitive elements accounted for approximately 83% of the genome (2.07 and 2.12 Gb) (Table S6). Notably, the proportion of long terminal repeat (LTR) retrotransposons was the highest, accounting for about 66.80% of the genome (Table S6). The BUSCO values of the protein coding gene structures were 91.00% and 91.60% (Table S4).

Chromosomes also have some special DNA regions that play important biological functions, including telomere and centromere. The telomere sequence are highly conserved in plants with 7-bp nucleotide unit repeat, and can protect chromosomes from fraying or tanging (Wang et al. 2023). Using TeloExplorer and CentroMiner module of quarTeT, we found the potential telomere and centromere regions of each chromosome (Table S7). The result showed that the majority of centromere region were located in the middle of chromosomes except chromosome 8, and the length ranged from 1,380,190 to 44,450,283 (Table S7). And only chromosome 1 presented telomere repeats at both ends with a total counts ranged in 1247 ~ 1826 (Fig. 1b). The CentIER software was also used to predict centromere region of chromosomes, and CentIER predicted lower length differences among centromere regions of differ chromosomes than quarTeT (Table S7). To verify the accuracy of the predicted centromere regeion on chromosome 1 (*Ac. Hap2*), we analysed the repeat density, gene density and Hi-C interactions. We discovered a location in the predicted centromere region of quarTeT with low repeat density, high gene density and high tandem repeat density compared with other regions on the chromosome (Fig. 1b). Meanwhile, the Hi-C interactions also showed that the location of centromere region was correct (Fig. 1b). However, centromere locations were completely predicted by bioinformatics, and the accurate positions need to be further validated.

### Haplotypic variation and allelic imbalance between *Ac. Hap1* and *Ac. Hap2*

To investigate sequence divergence of homologous chromosomes, we identified single nucleotide polymorphisms (SNPs), small insertions or deletions (indels), and other structural variation between the two haplotypes by the Synteny and Rearrangement Identifier (SyRI) tools. In comparison with *Ac. Hap1*, a total of 4,779,927 SNPs, 372,389 indels (< 50 bp), 1,589 translocations, 3,242 insertions (> 50 bp), 3,243 deletions (> 50 bp) and 100 inversions were identified in *Ac. Hap2* (Table S8). We further identified three large segment chromosomal inversions on chromosomes 10, 12, and 13, with lengths of 24.66 Mb, 22.53 Mb, and 37.41 Mb, respectively (Fig. 1c). The Hi-C signals at the inversion sites support the presence of these inversions (Figure S2), and these finding was further confirmed by examining the realignments of original HiFi reads against previously reported *A. catechu* genome assemblies (Figure S3-S4) (Yang et al. 2021b; Zhou et al. 2022a).

Allele exist in the same position on a pair of homologous chromosomes, and allele-specific expression could have profound effects on physiological processes and environmental adaptation (Zhang et al. 2021). By using MCscanX, we found 27,660 allelic gene pairs between homologous chromosomes and 27,599 gene pairs maintained high levels of coding sequence similarity, among which 20,335 (73.68%) alleles had the same coding sequence and 7,264 (26.32%) alleles had similar sequence (Fig. 1d). Based on the RNA-seq datasets of root, stem, leaf, and different part of flowers, 984 allelic gene pairs exhibited significantly allele-specific expression (TPM > 1, fold change > 2 and *P*-value < 0.05) (Table S9-S10). Among these differently expressed allelic gene pairs, 131 allelic gene pairs were exist in one tissue, 179 were exist in all tissues, and 674 were exist in multiple tissues (Fig. 1e). The highest number of tissue-specific alleles were found in different tissues of flowers, and stamen of male flower existed largest number (Fig. 1e). For instance, the *AcROMT* gene showed an obvious inconsistent expression pattern across seven different tissues of flowers and the *AcROMT* (*Ac. Hap1*) expression near to zero (Fig. 1f). The ortholog in *Vitis vinifera* encoded a resveratrol *O*-methyltransferase that was able to catalyze the biosynthesis of pterostilbene from resveratrol both in vitro and in planta (Schmidlin et al. 2008). We observed four insertions (4, 10, 11 and 77 bp) and one deletion (110 bp) in coding sequence between two haplotypes, which caused a low identity (69.25%) of amino acid sequence (Fig. 1f). Haplotype genomes can help understand the structural variations between allelic gene pairs that lead to allelic imbalance.

### Phylogenomic analysis of Arecaceae and ancient monocot karyotypes construction

**Fig. 2:**
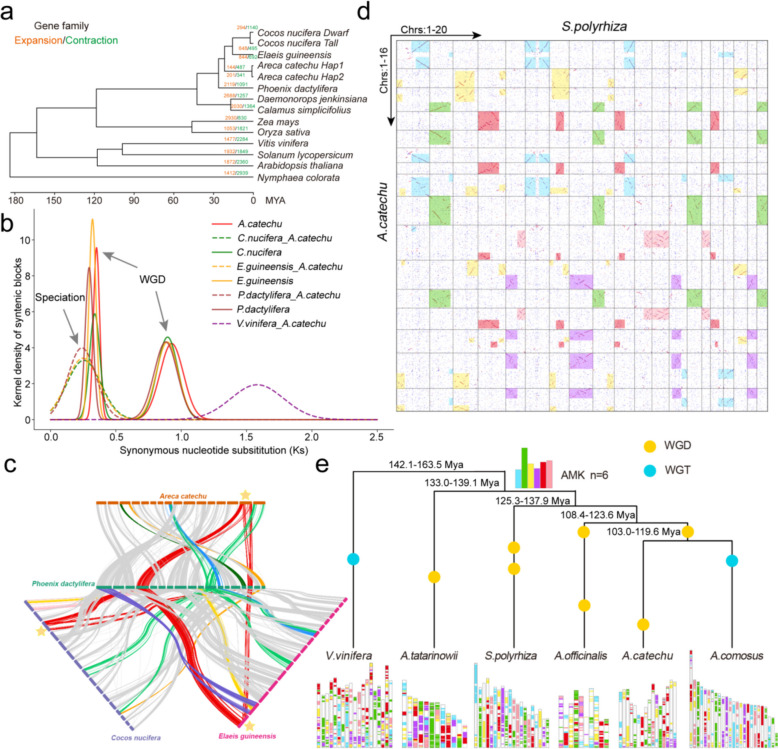
Analysis of species evolution and construction of ancestral monocot karyotypes (AMK). **a** Phylogenetic tree constructed through concatenation method by using protein sequence of twelve species. Orange and green represent expanded and contracted gene families, respectively. **b** *Ks* density distribution. The solid line represents comparisons of species themselves, and the dotted line represents comparisons between different species. **c** Collinearity between chromosomes among *Ac. hap1* and three other palm plants. **d** Dotplot between *Ac. hap1* and *S. polyrhiza.* Blocks with the same colors represent homologous chromosome fragments originating from the same ancestral chromosome. **e** Species phylogeny and reconstructed monocot proto-karyotypes. WGD and WGT are represented by yellow and blue dots, respectively

To investigate the phylogenetic placement of *A. catechu*, we collected protein sequences from twelve representative genomes and two *A. catechu* haplotype genomes for phylogenetic analysis, encompassing six palm plants and six other species (Table S11). Palm plants could be divided into two branches based on our construction of phylogenetic trees using 652 strictly single-copy orthologs. The first branch contains *Calamus simplicifolius* and *Daemonorops jenkinsiana,* and the second contains *Phoenix dactylifera*, *A. catechu*, *Cocos nucifera* and *Elaeis guineensis.* We estimate that *P. dactylifera*, *A. catechu*, *C. nucifera* and *E. guinenensis* differentiated from other palm plants approximately 40 million years ago (Mya), and *A. catechu* differentiated approximately 20 Mya (Fig. 2a). These data indicated that the genetic relationships between the four palm family species were closer than other species. A total of 29,031 *A. catechu* genes (84.79%) were clustered in 14,046 gene families, with 7,958 families (56.66%) shared among all 13 species and 10,633 families (75.70%) shared among 6 palm family species, 144 and 201 families underwent expansion, and 487 and 341 families underwent contraction (Fig. 2a). KEGG pathway enrichment analysis revealed that expansion gene families were significantly enriched in biological processes such as terpenoid, pantothenate and CoA biosynthesis (Figure S5). GO enrichment analysis revealed that contraction gene families were significantly enriched in molecular function such as UDP-glucosyltransferase activity and especially enriched in quercetin glucosyltransferase activity, which showed the significant reduction of flavonoid glycosyltransferases in *A. catechu* (Figure S5).

Given that WGDs contribute to genome size expansion and species evolution, we compared *A. catechu* with three other palm plants (*C. nucifera*, *E. guinenensis* and *P. dactylifera*) to analyze whether they had experienced specific WGD events. Our analysis found two overlapping *Ks* (~ 0.3 and ~ 0.9) peaks in the gene pairs of four species, indicating two WGD (~ 26.78 and ~ 80.34 Mya) events among the four palm family species (Fig. 2b). The value of the *Ks* peaks (~ 0.25) between palm plants were slightly smaller than the value of the second WGD *Ks* peak (~ 0.3), indicating that the four species underwent speciation in a short period of time following the second WGD event (Fig. 2b). Based on our previous studies of *C. nucifera* chromosomal evolution, the earliest WGD event (~ 80.34 Mya) was shared by multiple closely related species (*Musa acuminate* and *Ananas comosus*) and the later WGD event (~ 26.78 Mya) was palm-specific at present (Wang et al. 2021). Synteny analysis revealed substantial chromosomal regions with highly continuous gene collinearity between *A. catechu* and other palm family species (Figure S6). Using chromosome karyotype evolution analysis, we found that chromosome 4 and chromosome 16 of *P. dactylifera* were arranged as a single chromosome in the other three palm family plants (Fig. 2c). Overall, the evolution of the four palm family plants was slow, but included chromosomal fusion and separation.

The high-quality *A. catechu* genome and high collinearity between palm family plants make it possible for *A. catechu* to replace coconut and oil palm in reconstructing monocot ancestral karyotypes. Previous studies have constructed AMKs with 5, 6, and 10 chromosomes. Two monocotyledonous plants, *Spirodela polyrhiza* (*n* = 20) and *A. catechu* (*n* = 16), were used to reconstruct ancestral chromosomes by chromosome collinearity (Hoang et al. 2018). Using the dot matrix diagram approach for these two species, we identified six large gene collinear chromosomal fragments with a ratio of 4:4 between chromosomes (AcChrs:1–16 and SpChrs:1–20) (Fig. 2d). The intragenomic syntenic relationship showed that most chromosomes had a single homologous block (blue block: AcChr1, AcChr6 and AcChr8; green block: AcChr5, AcChr9 and AcChr12; red block: AcChr4 and AcChr7; yellow block: AcChr2) (Fig. 2d). Pink or purple blocks existed on same chromosome with other color blocks on *A. catechu* chromosome, but singly existed on multiple chromosomes of *S. polyrhiza* (pink block: SpChr11, SpChr12 and SpChr15; purple block: SpChr8 and SpChr19) (Fig. 2d). Considering that WGD could improve the preservation probability of ancient chromosome, we made inferences regarding a monocot ancestor possessing six chromosomes based on the six color blocks (Fig. 2d). Next, based on the color blocks distribution characteristics of the *A. catechu* chromosome, it was helpful to speculate the chromosome evolution of *A. catechu*. For example, according to the color blocks arrangement order of AcChr15 and AcChr16 (purple, blue and yellow), it indicated that the two chromosomes were directly generated by the same pre-chromosome after one WGD event, and the pre-chromosome had undergone chromosome fragment exchange before the WGD (Fig. 2d). In particular, AcChr1 exhibited high collinearity within the chromosome indicating that the chromosome was produced by the fusion of two highly homologous chromosomes after two WGDs (Fig. 2d). Based on the above results, we preliminarily inferred the chromosome evolution process of *A. catechu* from a monocot ancestor possessing six chromosomes (Figure S7). After undergoing the first WGD event (*n* = 12), *A. catechu* experienced two chromosomal separations and five chromosomal fusions, resulting in the formation of nine chromosomes from the monocot ancestors (Figure S7). Then, after the second WGD event (*n* = 18), one chromosome separation and three chromosome fusions resulted in 16 chromosomes (Figure S7). Using 25 chromosomes of *Ananas comosus* (1 WGD and 1 WGT) for validation, we found that six chromosomes of monocot ancestors had multiple single chromosomes collinear with them in *A. comosus* (Figure S8) (Ming et al. 2015). Based on the 6 ancestral chromosomes, using the collinearity of gene fragments between ancestral and species chromosomes, we inferred the origin and composition of each chromosome in the other species (Fig. 2e).

### *A. catechu* genome expansion is linked with retrotransposons

**Fig. 3:**
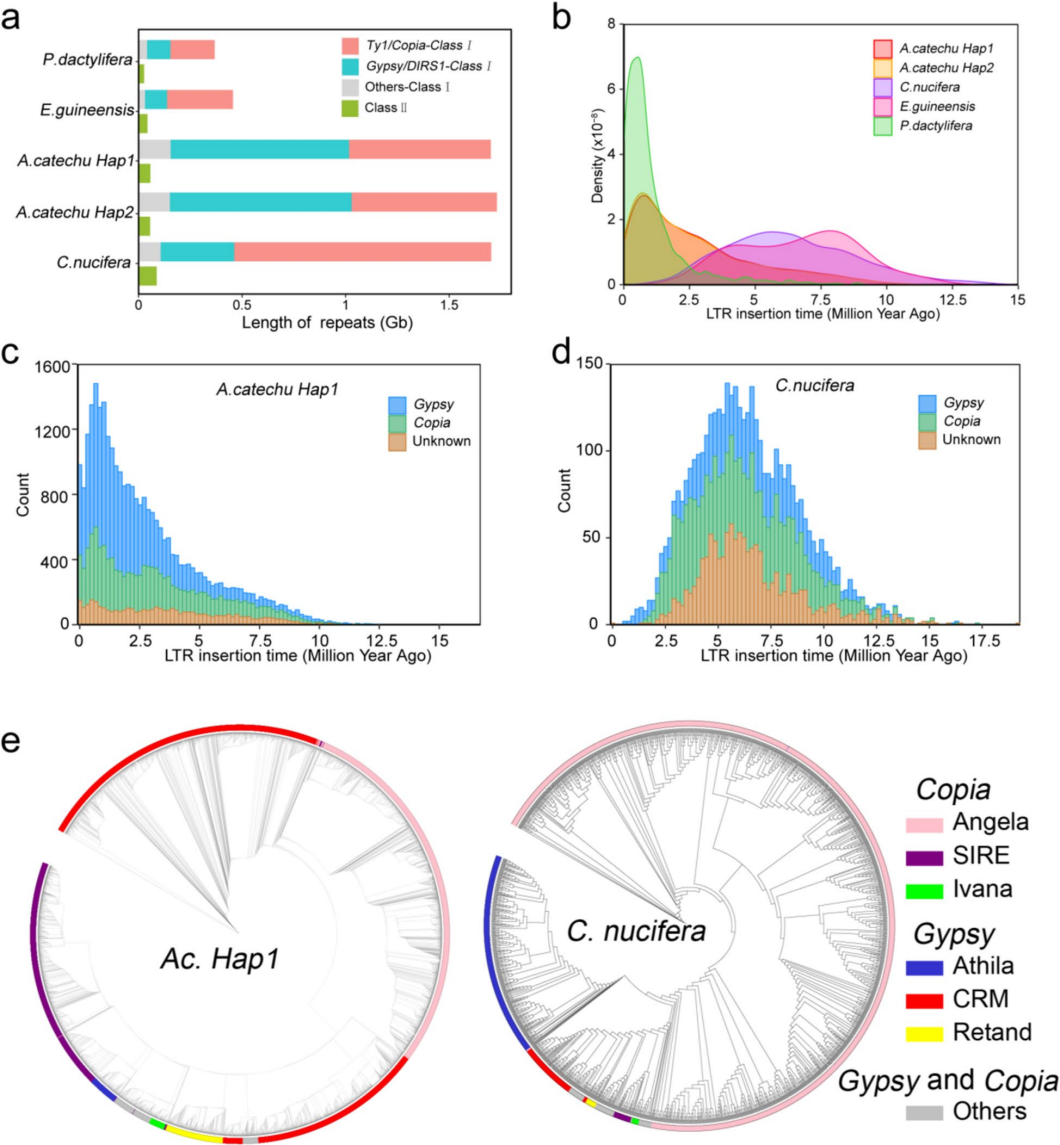
Comparative analysis of transposons in palm plants. **a** Sizes of Class I and Class II transposons on the genome of palm plants. **b** Density distribution curve of transposon insertion time. Red and yellow curves represent *A. catechu*; Purple represents *C. nucifera.* Pink represents *E. guineensis*; Green represents *P. dactylifera*. **c** Insertion time and counts of LTR-TRs (*Copia*, *Gypsy* and Unknown) in *Ac. Hap1*. **d** Insertion time and counts of LTR-TRs (*Copia*, *Gypsy* and Unknown) in *C. nucifera*. **e** The maximum likelihood phylogenetic tree of all *Copia* and *Gypsy* in *Ac. Hap1* and *C. nucifera* at whole genome-wide

Large plant genomes usually had a high proportion of repetitive sequence elements, e.g. ~ 74% in *Cycas panzhihuaensis* (genome size = 10.5 Gb), ~ 84% in *Cocos nucifera* (genome size = 2.40 Gb) and ~ 85% in *Ceratopteris richardii* (genome size = 7.46 Gb) (Liu et al. 2022; Marchant et al. 2022; Wang et al. 2021). Because *P. dactylifera*, *A. catechu*, *C. nucifera* and *E. guinenensis* underwent common WGDs, repeat sequence insertion may be the main factor driving differences in genome size (Zou et al. 2023). Repeat sequences in *E. guinenensis*, *C. nucifera*, *P. dactylifera* and *A. catechu* account for 58.59% (0.45 Gb), 83.14% (1.99 Gb), 45.27% (0.69 Gb), 84.40% (*Ac. Hap1*, 2.11 Gb), and 84.64% (*Ac. Hap2*, 2.16 Gb) of the entire genome (Fig. 3a and Table S12). LTR-RTs form the largest proportion of all repetitive sequences (Figure S9). In contrast to the other three species, the proportion of *Gypsy* (~ 34%, 0.86–0.87 Gb) is higher than that of *Copia* (~ 27%, 0.68–0.70 Gb) in *A. catechu* genome (Fig. 3a and Figure S10). The insertion of LTR-RTs occurred within the last 1 million years (Fig. 3b). For *A. catechu*, the insertion of LTR-RTs began around 10 Mya and burst within 2.5 Mya (Fig. 3b). This differs from the evolutionary history of *P. dactylifera*, in which most transposons were inserted within the last 2 million years. The transposon insertion patterns of *C. nucifera* and *E. guinenensis* suggests that transposon insertion was a continuous process in the two species (Fig. 3b).

The number of complete LTR-RTs recognized in the *A. catechu* genome (28,978 and 29,990) far exceeds that of *C. nucifera* (4120), *P. dactylifera* (3011), and *E. guinenensis* (72). The number of insertions of *A. catechu Gypsy* within the last 2.5 million years is higher than that of *Copia* (Fig. 3c). In contrast to *A. catechu*, the number of *Copia* and other types of LTR insertions in *C. nucifera* varies more than that of *Gypsy* and peaked around 6 Mya (Fig. 3d and Figure S10). In order to understand the evolution of LTR-RTs and the driving force of genome size in palm plants, we conducted a more detailed classification of all *Copia* and *Gypsy*, which identified at the whole genome wide (Fig. 3e). By comparing *Ac. Hap1* with *C. nucifera*, the extensive amplification of Angela (*Copia*) was the main driving force behind the increased genome size of *C. nucifera,* and the extensive amplification of CRM (*Gypsy*), Angela (*Copia*) and SIRE (*Copia*) jointly promoted size changing of *A. catechu* genome (Fig. 3e)*.* Meanwhile, the gene region coverage length of *E. guinenensis* and *P. dactylifera* was less than 100 Mb, while the total length of gene regions in *C. nucifera* and *A. catechu* exceeded 130 Mb (Figure S11). The average length of gene introns did not change significantly with increasing average gene length, and patterns of average intron length are consistent with changing gene lengths, indicating that differences in gene length were mainly caused by the difference in intron length (Figure S11). The insertion of transposons increased the genome size of *A. catechu* and *C. nucifera*.

### Gene-metabolite network associated with medicinal ingredients biosynthesis in *A. catechu*

**Fig. 4:**
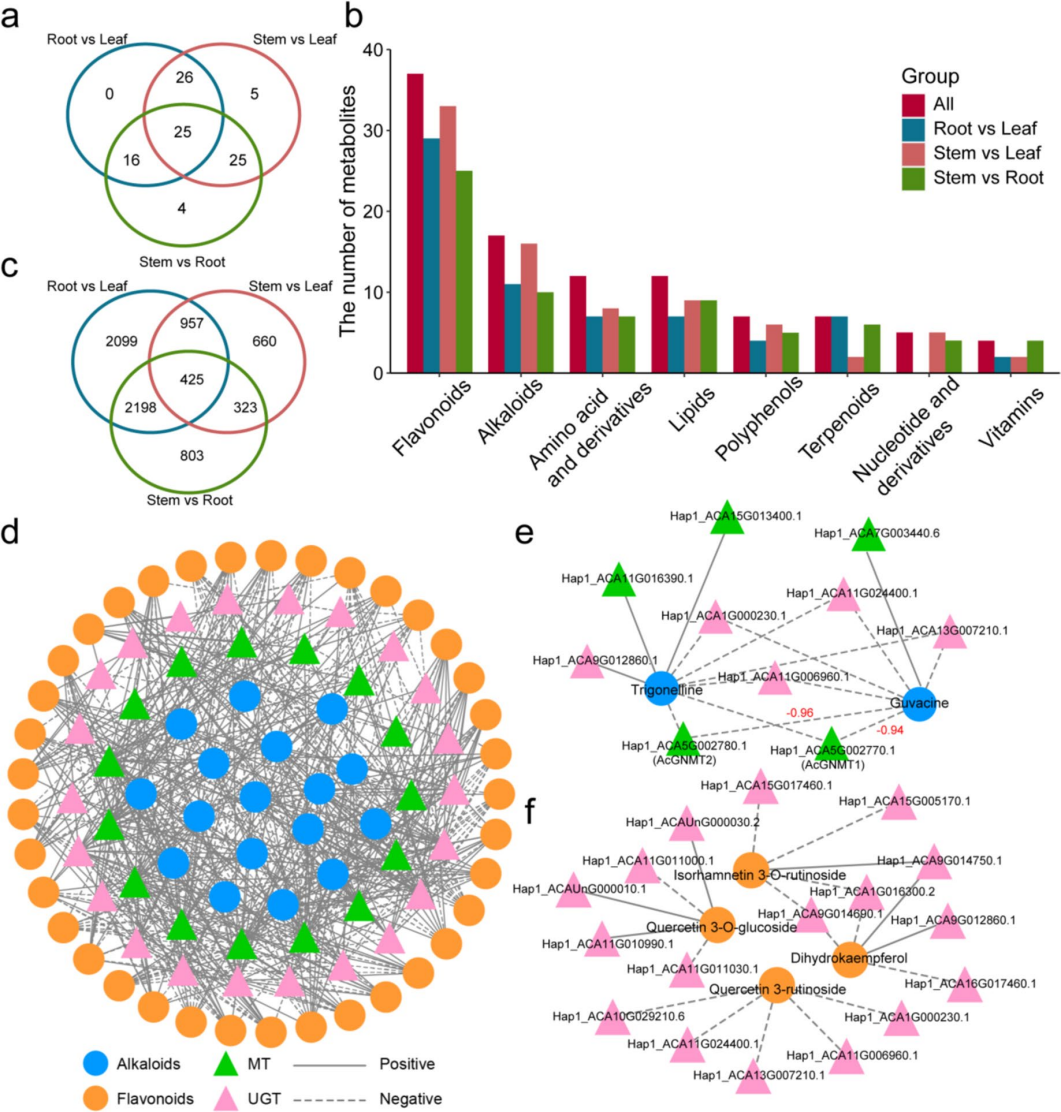
Comparative analysis of the *A. catechu* metabolome and transcriptome between different tissues. **a** Venn diagram showing the overlap between differentially accumulated metabolites (DAMs, VIP > 1 and |log_2_FoldChange|> 1). **b** Statistical analysis of DAMs of eight metabolite classes. **c** Venn diagram showing the overlap between the up- and down-regulated differentially expressed genes (DEGs, FDR < 0.05 and |log_2_FoldChange|> 1). **d-f** Interaction network indicating associations between medicinal ingredients and transferases. Blue circles represent alkaloids; orange circles represent flavonoids; green triangles represent MTs; pink triangles represent UGTs; solid lines represent positive correlation and dashed lines represent negative correlation

We conducted a series of comparative analysis among three tissues (root, stem, and leaf) by using metabolomic and transcriptome data (Table S9-S10 and Table S13). Based on metabolomic data comparison, we screened 101 differentially accumulated metabolites (DAMs), and the highest DAMs were observed between stem and leaf (Fig. 4a and Table S14). Among all DAMs, there were 37 flavonoids and 17 alkaloids, including the *A. catechu* specific alkaloid guvacine (Fig. 4b and Table S14). Then, a comparison of transcriptome data between tissues revealed a total of 7,464 differentially expressed genes (DEGs), with 425 are commonly DEGs (Fig. 4c and Table S15). In detail, we found 2,305, 3,936, and 1,524 up-regulated genes and 2,952, 3,363, and 1,870 down-regulated genes by comparing root and stem, root and leaf, and stem and leaf, respectively (Figure S12).

KEGG enrichment analysis showed that between-tissue DEGs were enriched in the phenylpropanoid and flavonoid biosynthesis pathways and in terpenoid backbone biosynthesis (Figure S13). In addition, GO enrichment analysis showed that DEGs were enriched in the phenylpropanoid and terpenoid biosynthesis processes and in phenylpropanoid metabolism (Figure S14). These results suggest that there may be tissue differences in the biosynthesis of bioactive substances such as flavonoids and terpenoids in *A. catechu*.

Pyridine alkaloids and glycosylated flavonoids have significant biological activities. In order to identify the MTs and UGTs that were involved in arecoline and flavonoid biosynthesis, we identified MTs and UGTs in the *A. catechu* genome. 56 MTs and 79 UGTs were identified in *Ac. Hap1*, and 55 MTs and 84 UGTs were identified in *Ac. Hap2* (Figure S15-S16 and Table S15-S17). Further screening identified 41 DEGs (15 MTs and 26 UGTs) from *Ac. Hap1*. Using calculations of the correlation between DEGs and DAMs, we constructed a correlation network containing 54 DAMs (17 alkaloids and 37 flavonoids) and 37 DEGs (15 MTs and 22 UGTs) (Fig. 4d and Table S18). Guvacine and trigonelline were negatively associated with two tandem MTs (*Hap1_ACA5G002770.1* and *Hap1_ACA5G002780.1*) on chromosome 5 (Fig. 4e). The protein sequences of the two genes were highly similar, with a homology of 96.79%. The two MTs identified on *Ac. Hap1* had corresponding tandem genes (*Hap2_ACA5G002750* and *Hap2_ACA5G002760*) on *Ac. Hap2*, and were conserved on gene structure and protein sequence between two haplotypes. Phylogenetic analysis of MTs revealed that the tandem genes and the reported nicotinate *N*-methyltransferase (NANMT) were on the same branch (Figure S17). ZmNANMT1 and AtNANMT1, which have been reported in maize and Arabidopsis, can catalyze trigonelline synthesis from nicotinate (Li et al. 2023b; Li et al. 2017). Because nicotinate and trigonelline have chemical structures similar to arecoline, it has been speculated that they are the precursor to arecoline biosynthesis. The two tandem genes (*Hap1_ACA5G002770.1* and *Hap1_ACA5G002780.1*) and their homologous genes may be involved in arecoline biosynthesis. Because flavonoids have similar structures, flavonoid UGTs often use multiple types of flavonoids as substrate. Figure 4F shows that isorhamnetin 3-*O*-rutinoside was negatively associated with a gene (*Hap1_ACA15G017460.1*). Previously, we used in vitro experiments to show that it can catalyze glycosylation modification of kaempferol and chrysin (Lai et al. 2023), suggesting that other genes associated with these flavonoids in the subnetwork can be used as candidate genes (Fig. 4f).

### The biosynthesis pathway of arecoline and the diversity of flavonoid glycosylation in vitro

**Fig. 5:**
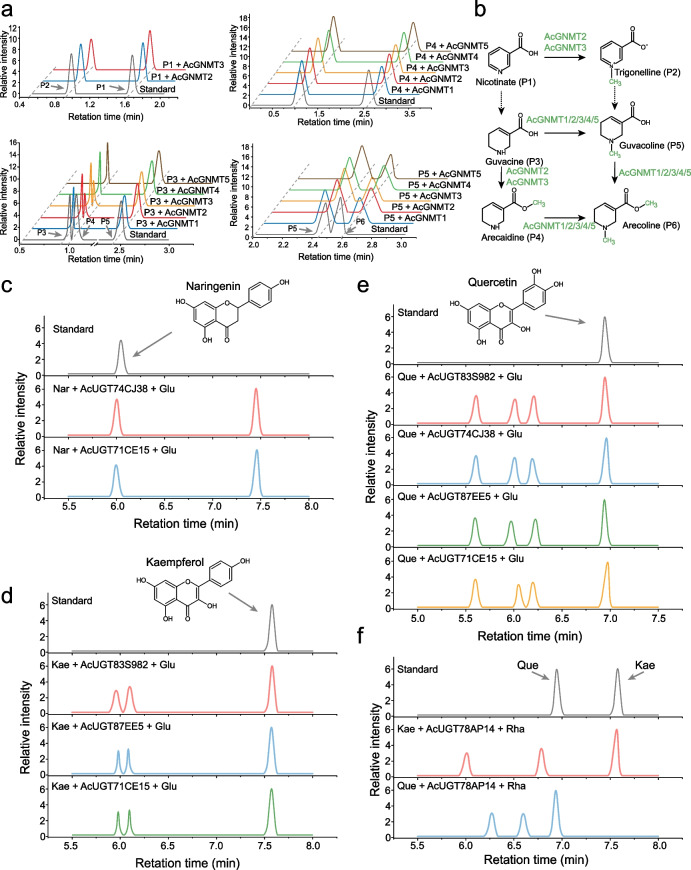
Function identification of AcGNMTs and AcUGTs in *A. catechu*. **a** Biosynthesis pathway of arecoline in *A. catechu*. Solid arrows indicate the identified steps, whereas the dashed arrows show the hypothetical steps. **b** Analysis of AcGNMT activity in vitro. High-performance liquid chromatography (HPLC) chromatograms for recombinant AcGNMTs with nicotinate, guvacine, guvacoline, and arecaidine. **c-f** Analysis of AcUGT activity in vitro. High-performance liquid chromatography (HPLC) chromatograms for recombinant AcUGTs with naringenin (**c**), kaempferol (**d, f**), and quercetin (**e, f**)

Compared with other palm plants, the diverse specific alkaloids of *A. catechu* mainly include arecoline, arecadine, guvacoline, and guvacine. The four alkaloids have similar molecular structures, and it is possible that they can be inter-converted through *O*-methyltransferase and *N*-methyltransferase, with guvacine being the most upstream metabolite. The similarity between the tandem genes *AcGNMT1* and *AcGNMT2*, which we found to form collinearity networks (Fig. 4e), exceeds 90%, and their homologous gene *NANMTs* have been reported previously (Li et al. 2023b; Li et al. 2017). To verify whether the two tandem genes are involved in the biosynthesis of arecoline, we conducted a series of in vitro experiments using guvacine, guvacoline, and arecadine as substrates, and SAM as methyl donor. In vitro experimentation revealed that only AcGNMT2 had the ability to facilitate the conversion of nicotinate to trigonelline, but both AcGNMT1 and AcGNMT2 could catalyse the conversion of guvacine (P3) to arecoline (P6) through *N*-methylation and *O*-methylation (Fig. 5a). In addition to *AcGNMT1* and *AcGNMT2*, we also characterized three other *AcGNMTs*, among which *AcGNMT3*/*4* in the same branch with reported caffeic acid *O*-methyltransferases and *AcGNMT5* (*Hap1_ACA13G008210.1*) was one of the tandem *MTs* on chromosome 13 (Figure S15 and Figure S17). AcGNMT3 had a function similar to AcGNMT2, and AcGNMT4/5 had similar functions with AcGNMT1 (Fig. 5a and Figure S18). Overall, five AcGNMTs were validated as participating in arecoline biosynthesis (Fig. 5b).

Flavonoids have similar molecular structures, and UGT is often reported to catalyze multiple types of flavonoids (Lai et al. 2023). In the genes-metabolites correlation network, multiple UGTs were associated with flavonoids, including a reported UGT (*Hap1_ACA15G017460.1*) contained in this network (Figure S13) (Lai et al. 2023). Using the phylogenetic tree and this network of the UGT family, a rhamnosyltransferase (*AcUGT78AP14*) and four glucosyltransferases (*AcUGT71CE15*, *AcUGT74CJ38*, *AcUGT87EE5* and *AcUGT83S982*) were validated.

In vitro, AcUGT74CJ38 can modify naringenin and quercetin with glucose residues, while AcUGT71CE15 show activity towards the three flavonoids (Fig. 5c-e and Figure S19). Both of AcUGT87EE5 and AcUGT83S982 showed activity only towards kaempferol and quercetin (Fig. 5d-e and Figure S19). AcUGT78AP14 can modify kaempferol and quercetin with the sugar donors of UDP-Rhamnose, and can catalyze the formation of quercetin *C*-rhamnoside (RT = 6.34 min) (Fig. 5f and Figure S19).

## Discussion

Given the economic importance of palm plants and their importance in species phylogeny, we assembled two haplotype-resolved genomes of *A. catechu* (*Ac. Hap1* and *Ac. Hap2*) using HiFi and Hi-C data. Considering the assembly difficulty caused the large genome size, N50 of the haplotype genome of *A. catechu* exceeds 30Mb, which is 100 times higher than the previously reported genomes (Yang et al. 2021b; Zhou et al. 2022a). Assembling haplotype-resolved genomes at the chromosome scale can help us understand genetic differences between homologous chromosomes (Jiang et al. 2024; Zhang et al. 2024). Although the two haplotype chromosomes exhibit high collinearity, there are still many structural differences, especially the three large segment inversions on chromosome 10, 12, and 13, which directly affect DNA sequence evolution and gene numbers. By comparing *Ac. Hap1* with *Ac.Hap2*, we found that they had 8.50 (*Ac. Hap1*) and 49.88 Mb (*Ac. Hap2*) specific chromosomal segments, which contained 111 and 1146 genes respectively (Figure S20). At the same time, the coding regions of 649 (*Ac. Hap1*) and 1,217 (*Ac. Hap2*) low expressed genes had SVs (deletions, insertions, inversions and translocations) (Figure S21). In general, the gene numbers differences between *Ac. Hap1* and *Ac. Hap2* were due to specific chromosomal fragments and SVs.

By comparing the *A. catechu* genome with those of other plants, such as water lily, *C. nucifera*, *P. dactylifera*, and *E. guinenensis*, phylogenetic analysis clearly reveals the evolutionary position of *A. catechu* in monocot plants and its evolutionary relationships with other palm plants (Zhang et al. 2020). Thanks to the increasing availability of high-quality genome sequences, the phylogenetic relationships between palm plants are becoming clear. We inferred from the Ks plot that *A. catechu* had two WGDs during its evolution. This is a history that is shared with other palm plants (*C. nucifera*, *P. dactylifera*, and *E. guinenensis*) prior to speciation and is consistent with previous *C. nucifera* genome research (Wang et al. 2021). Chromosome fusion or division could lead to changes in genetic information on chromosomes, such as in *Saccharum spontaneum*, and we also found the variation in palm plants (Zhang et al. 2022). By comparing sequence consistency the distribution of homologous genes between *A. catechu* and other palm plants, we found that the chromosomes underwent fusion or division following WGDs. For example, chromosomes 4 and 16 in *P. dactylifera* had been fused into a single chromosome in other palm plants, which was corresponds to chromosome 15 in *A. catechu* (Fig. 2c). The different fragments of AcChr15 respectively corresponded to multiple chromosomes in *S. polyrhiza*, and we speculated that the chromosome contained fragments of different ancestral chromosomes, most of which came from the purple and yellow blocks (Fig. 2d). Given the highly collinear arrangement of homologous genes on palm plant chromosomes, *A. catechu* can replace *E. guinenensis* and *C. nucifera* as a reference template for constructing AMKs (Wang et al. 2021; Murat et al. 2017). We constructed the AMKs of 6 chromosomes to help clarify the evolutionary trajectory of existing chromosome formation in *A. catechu* and each chromosome was highly collinear with multi chromosomes of pineapple (*n* = 25), where purple and yellow blocks were also supported by multiple chromosomes (Figure S8). Therefore, we speculated that pdChr4 and PdChr6 were fused to generate AcChr15 in the common ancestor of other palm plants after the differentiation of *P. dactylifera*. However, multiple complex chromosomal variations prevented us from understanding the cause of the difference in chromosome number between *P. dactylifera* (*n* = 18) and other palm plants (*n* = 16) (Fig. 2c).

Species with larger genomes may exhibit higher adaptability and evolutionary potential, and the *A. catechu* genome is the largest of palm plants that have been sequenced. According to previous work, the genomes of *A. catechu* and *C. nucifera* exceed 2.4 Gb in size, while *E. guinenensis* and *P. dactylifera* are only 1.54 Gb and 0.77 Gb. Considering that they experienced the same WGDs, the main factor contributing to differences in genome size is transposon insertion following species differentiation. In contrast to *C. nucifera* and most other species, *Copia* is inserted into the *A. catechu* genome more frequently than *Gypsy*. With the expansion of genome size, increased intron lead length increased total gene length. Although research in Chinese pine suggests that super long introns may have higher expression activity, super long exons can cause difficulty in gene prediction (Niu et al. 2022). Here, we found that the tandem genes *AcGNMT1* and *AcGNMT2* have ultra-long introns, exceeding 17kb and 6 kb, which caused incorrect gene annotation in previous studies. High-quality assembly and precise gene annotation of large genomes have always been challenging.

As the leader of the “Four Great Southern Medicines”, seeds from *A. catechu* are used in traditional Chinese medicine. The most important active ingredients are alkaloids, mainly arecoline and guvacoline. Previous work has elucidated various alkaloid structures in *A. catechu*, but the specific synthesis pathways of arecoline have not been thoroughly studied (Wu et al. 2022). Based on similarity in chemical structure, is seems likely that nicotinate is the precursor of arecoline, and methyltransferase and reductase form the key genes in the pathway. By using reported MTs as reference, we identified 56 (*Ac. Hap1*) and 55 (*Ac. Hap2*) MTs across the entire genome as well as MTs exhibiting obvious tandem clustering on chromosome 13, which help us focused on *AcGNMT5*. In addition, flavonoids such as quercetin and kaempferol have various biological activities and are ubiquitous in plants (Lai et al. 2023). These flavonoids are most frequently modified by glycosylation. As a result, we identified 79 (*Ac. Hap1*) and 84 (*Ac. Hap2*) UGTs in a gene family manner from the *A. catechu* genome. Then we constructed a correlation network between DAMs and DEGs to identify candidate genes involved in the biosynthesis of medicinal ingredients. Here, we found in vitro AcGNMTs substrate diversity, and conversion between multiple specific alkaloids in *A. catechu* can be completed with one AcGNMT. However, AcUGTs located on different chromosomes that exhibit the same catalytic function, such as AcUGT87EE5, AcUGT83S982, and AcUGT71CE15 can catalyze kaempferol and quercetin to produce either two or three glycosylation products, illuminating the molecular mechanism driving the diversity of flavonol glycosides in *A. catechu.* As a perennial woody plant, *A. catechu* genes can serve as an ideal model for studying the biosynthesis of medicinal ingredients.

The two haplotype-resolved genomes reported in this study provide insights into homologous chromosomal genetic differences, AMK construction, and the metabolic synthesis of bioactive compounds. Considering the medicinal value of *A. catechu* and the difficulty of genetic modification like kiwifruit (Li et al. 2024), this work provides a basis for the use of important tropical medicinal resources and breakthrough of functional genomic studies.

## Methods

### Plant materials and sequencing data

Materials of *A. catechu* were collected from the Agricultural Science Experimental Base of Hainan University, Haikou, China. The transcriptome and metabolome data of leaf, stem, and root were collected from our previous study (Lai et al. 2023). For Circular Consensus Sequencing (CCS), genomic DNA was extracted from frozen A. catechu mixed tissue samples using the CTAB method. A 15 Kb DNA SMRTbell library was constructed and sequenced on the PacBio Sequel II platform. The Pacbio HiFi reads were generated from the raw data (PacBio subreads). The Hi-C library was sequenced using the Illumina Nocaseq/MGI-2000 platform.

### Estimation of the genome heterozygosity

Before assembling the reference genome, we used 75 Gb HiFi data to estimate the genome heterozygosity of *A. catechu*. Then, K-mer frequency (k = 21) was calculated using Jellyfish (v2.3.0) and the genome heterozygosity was 0.50% estimated using findGSE (Sun et al. 2018).

### Genome assembly and quality assessment

We employed the CCS tool embedded within SMRT Link (min-passes: 10) to generate HiFi reads from the raw PacBio subreads. The raw Hi-C reads were quality controlled through fastqc and low-quality reads are filtered by fastp (v0.23.4). Two haplotypes were assembled using Hifiasm (v0.16.1) under the HiFi + Hi-C mode (Cheng et al. 2021), with the parameters of -l3 and –hom-cov 28. After completing the initial assembly, we scaffolded contigs using cleaned Hi-C sequencing data using Juicer (v1.6) (Durand et al. 2016) and 3d-DNA (v 190716) pipelines (Dudchenko et al. 2017). After the scaffolding, Juicerbox (v1.11.08) was used to visualize Hi-C diagrams and manually fix incorrect connections and clustered contigs (Robinson et al. 2018). Finally, the manually corrected results were reassembled into the final genome using 3d-DNA. Merqury software was used to calculate the consensus quality value and BUSCO (embryophyta_odb10) was used to evaluate the assembly completeness (Rhie et al. 2020).

### Repeat elements annotation, gene prediction and functional annotation

Annotations of protein-coding genes were performed using ab initio prediction, protein homology, and transcriptome evidence. Augustus (v3.2.3) (Stanke et al. 2006), GlimmerHMM (v3.0.4) (Majoros et al. 2004), and SNAP (v2006-07–28) (Korf 2004) were used for ab initio prediction. Protein sequences of *Arabidopsis thaliana*, *Oryza sativa*, *Cocos nucifera*, *Elaeis guinensis*, and *Phoenix dactylifera* were selected and aligned to the assembly by Exonerate (v2.4.7) (Slater et al. 2005). RNA-seq data were aligned to the genome and assembled by HISAT2 (v2.2.1) (Kim et al. 2015) and StringTie (v2.2.1) (Pertea et al. 2015). EVidenceModeler (v1.1.1) (Haas et al. 2008) was used to generate the gene models by integrating the evidences from the above three methods. Finally, PASA pipeline (v2.5.2) (Haas et al. 2003) was used to predict UTRs and alternative splicing events.

Repeat elements in the *A. catechu* genome were identified by combining the de novo prediction and the homology-search based methods. RepeatModeler (v2.0.3) used for de novo prediction of repeat sequences in the *A. catechu* genome. LTRs were identified by LTR_finder (Xu et al. 2007) and LTRHarvest (Ellinghaus et al. 2008) and further integrated by LTR-retriever (v2.9.0) (Ou et al. 2018) to obtain a database of *A. catechu* genome LTRs. Repbase serves as an annotated repeat dataset based on homology (Bao et al. 2015). RepeatMasker (v4.1.2) were used to identify repetitive sequences with the new database composing of repeat sequences, which were recognized by Repbase, LTR_retriever, and RepeatModeler. The function of protein coding genes were annotated by doing blast (e-value 1e-5) sequence alignment against Databases such as InterProScan (Jones et al. 2014), Swiss-Prot (Duvaud et al. 2021), and TrEMBL (Bairoch et al. 2000).

### Telomere detection and centromere localization

Telomere and centromere was identifed by the quarTeT (Lin et al. 2023) toolkit and CentIER (Xu et al. 2024) with default parameters. The continuous and high-frequency regions were regarded as candidate centromere regions. At last, combine the result of gene density, TE number and Hi-C interaction with candidate regions to predict the most likely centromere location.

### Identification of SNPs and structural variations

The SNPs, indels (< 50 bp) and structural variations (> 50 bp) were identified using SyRI (v 1.6.3) (Goel et al. 2019). The plotsr (v1.1.1) (Goel et al. 2022) was used for visualization. To verify the existence of several major structural variations, the HiFi reads were aligned to the assembled genome using Minimap2 (v2.24) (Nguyen et al. 2024), and the read alignments at the breakpoints of structural variations were manually checked using Integrative Genomics Viewer (IGV). Simultaneously, the Hi-C reads were aligned to the genome using BWA (v0.7.17) (Li et al. 2010), and the interaction at structural variations was manually checked using Juicerbox.

### Determination of allele-specifc expression

Synteny blocks between *Ac. Hap1* and *Ac. Hap2* were identified by MCScanX (Wang et al. 2012) and the sequence similarity of coding sequence were identifed using BLAST. A total of 12 tissues RNA-seq datasets were aligned to the two haplotype genomes and then used to calculate the gene expression values using the method described below. Allele-specific expression were determined using the criterion that the log_2_(fold change) > 1 or < -1, TPM > 1 and *P-*value < 0.05. The *P*-value was calculated by python script.

### Phylogenomic analysis

We collected protein sequences from *A. catechu* and 11 other genomes (Table S11). For genes with selective splicing variations, the longest transcript is retained in the analysis. OrthoFinder (v 2.5.4) (Emms et al. 2019) was employed for the identification of orthologous gene families across plant species. Through gene family analysis, a total of 652 single copy orthologous genes were obtained. MUSCLE (v3.8.31) (Edgar 2022) was used to align the protein sequences of single copy orthologous genes from 12 species, and using RaxML (8.2.12) (Stamatakis 2014) was used to construct a phylogenetic tree based on multiple sequence alignment results. The MCMCTree program in PAML (v4.9) (Yang 1997) was used to estimate the divergence time of all the 12 species, with previously released calibration time (109.8–124.4 Mya for *A. thaliana* and *V. vinifera*, 41.4–51.9 Mya for *O. sativa* and *Zea mays*) (Kumar et al. 2022). CAFÉ (v5.1) (Mendes et al. 2021) was used for inferring the expansion and contraction of gene families based on phylogenetic tree and gene family analysis results. Families with a gene ≥ 200 for one species and a gene ≤ 2 for all other species are discarded. The R package ClusterProfiler (v4.9.1) (Yu et al. 2012) is used to GO and KEGG enrichment analysis of expansion and contraction gene families.

### Identification of WGD and construction of AMK

To determine the WGDs involved in species, we used WGDI (Sun et al. 2022) to identify gene collinear blocks within species. We used WGDI to calculate the Ks between homologous gene pairs of five species, *C. nucifera*, *E. guinensis*, *P. dactylifera*, *V. vinifera*, and *Ac. Hap1*, and determined the number of WGDs in *A. catechu* and species differentiation time. The nucmer in Mummer (v3.32) (Kurtz et al. 2004) is used for chromosomes alignment among palm family species, and the whole genome collinearity results were visualized using NGenomeSyn (v1.41) (Zhou et al. 2022b).

For the construction of the ancestral genome, we firstly used WGDI to draw a dotplot between *Ac. Hap1* and *S. polyrhiza* to identify similar fragments on chromosomes. Then, we locate the large fragment region that matches the rate of 4:4, inferring that it originates from the same ancestral chromosome,and labeled with the same color. Finally, ancestral chromosomes were constructed based on the arrangement order of *A. catechu* genes on chromosomes. In order to display the sources of each chromosome fragment, we use MCScanX (Wang et al. 2012) to identify the chromosomal collinear regions between AMK and other species. Use local R script to draw the distribution of ancestral chromosome fragments on others species’s chromosomes.

### Calculation of LTR-RT insertion time

The complete annotation information for LTR-RT was obtained from the result of LTR_retriever, which had mentioned above. BEDTools (v2.27) (Quinlan et al. 2010) are used to obtain 5'/3' LTR sequences, and MUSCLE (v3.8.1551) is used to align the LTR sequences. Finally, use R package ape to calculate DNA genetic distance. We are estimate the insertion time based on T = D/2 μ, where D is the differentiation rate, μ It is the neutral mutation rate (5.6e-10).

For more detailed classification, TEsorter (v1.4.6) was used to classify all *Copia* and *Gypsy* sequences, while the sequences of RNaseH, integer, and reverse transcriptase were simultaneously used to construct phylogenetic trees. After sequence alignment using MUSCLE (v3.8.1551), trimAl (v1.2) (Capella-Gutiérrez et al. 2009) was used to trim alignment result. Finally, construct a protein tree using FastTree (v2.1.11) (Price et al. 2010). ITOL is used to beautify the phylogenetic tree.

### Metabolome and transcriptome data analysis

The criteria for identifying DAMs are fold change > 2 or < 0.5, and VIP > 1. The raw data of RNA-seq was filtering low quality reads and trimming adaptors by Fastp (v0.23.4) software with default parameters. HISAT2 (v2.2.1) was used to align Clean data to *A. catechu* haplotype-resolved genome. SAMtools (v1.9) was used to sort the results. Finally, featureCounts (v2.0.0) (Liao et al. 2014) used for quantification, and local R scripts were used to standardize the quantitative results using TPM (Transcripts Per Million). DEGs were calculated using the DESeq2 (v1.32.0) R package. The screening criteria for DEGs are Log_2_(Fold Change) > 1 or < -1, and adjusted *p*-value < 0.05.

### Construction of a correlation network between metabolites and genes

For the identification of MTs, we collected protein sequences of reported MTs from 14 different species. Then, DIAMOND (v2.0.4.142) (Buchfink et al. 2015) was used to blast all protein sequences of *A. catechu* with repoted MTs to obtain candidate MTs. And the HMM profile of the UGT domain (PF00201) was searched against all protein sequences to identification AcUGTs (Li et al. 2023a). In order to obtain high confidence results, we further screened differentially expressed MTs and UGTs with the lowest expression level > 1 and the highest expression level > 5 in all tissues. We used Python script to calculate the correlation between DAMs and DEGs, and each metabolite retained the top five genes with the highest correlation (|Correlation coefficient|> 0.8). Finally, we use Cytoscape (v3.7.1) to construct the correlation network.

### In vitro validation of candidate genes

The gene CDS sequence was amplified by high fidelity PCR enzyme (TOYOBO). By homologous recombination, we constructed the PMAL vector that was used for the protein expression. The recombinant protein was induced with 1 mM Isopropyl-β-D-thiogalactoside in a shaker overnight (160 rpm and 16 °C) to obtain purified protein. Next, the purified protein of MTs and GTs was prepared for in vitro activity assays. The reaction system of MTs activity assays in vitro contained 1 µl 0.4 mM substrate (nicotinate, guvacine, guvacoline, or arecaidine), 1 µl 2 mM SAM, 2.5 mM MgCl_2_, 10 µl 100 mM Tris–HCl buffer (pH = 7.5) and 500 ng purified protein, and the reaction was incubated at 30 °C overnight (Nett et al. 2020). The enzyme assays of UGTs were performed in a volume consisting of 1 µl 1 mM kaempferol, quercetin, and naringenin as substrates, 1 µl 0.2 mM UDP-glucose/UDP-rhamnose, 1 µl 2.5 mM MgCl_2_, 10 µl 100 mM Tris–HCl buffer (pH = 7.5), and 500 ng purified protein, and the volume incubated at 37 °C for an hour (Lai et al. 2023). The mixture was vortexed with 50 µl pre-cooled methanol to terminate the reaction and was centrifuged for 10 min (4 °C and 12,000 rpm) to separate the organic phase. Finally, 50 µl supernatant of the mixture was used for LC–MS analysis.

The reaction mixture was analyzed using high-resolution Agilent 6560C Ion-Mobility LC/Q-TOF. HPLC conditions for the analysis of arecolines and flavonoids were as follows: column, Waters Acquity UPLC HSS T3 C18 column (100 × 2.1 mm); injection volume was 2 μl; flow rate was 0.35 ml/min; the temperature was 40 °C; The mobile phase consisted of 0.1% formic acid in water (phase A) and 5 mmol ammonium formic acid in acetonitrile (phase B). the gradient of solvent A: solvent B was 100:0 for 0 min, 95:5 for 2 min, 5:95 for 12 min, 5:95 for 13 min, 100:0 for 13.1 min, 100:0 for 17.0 min. An Ion mobility Quadrupole-TOF mass spectrometer (Agilent Technologies) for qualitative analysis using auto-MS/MS mode in positive. The ion source main parameters of the Gas temperature 250 ℃; Gas flow 8 l/min; Nebulizer 35 psi; sheath gas temperature 375 ℃; sheath gas flow 11 l/min; capillary 3500 V; Nozzle Voltage 0 V; Fragmentor 400 V; Skimmer1 65 V; OCT RF 750 V. For the auto MS/MS mode, the MS scanning mass range was from m/z 50 to m/z 1500, MS scan rate 2.0 spectra/sec; the MS/MS scanning mass range was from m/z 50 to m/z 1500, MS/MS scan rate 3.0 spectra/sec; isolation width MS/MS narrow (~ 1.3 amu); fixed collision energies 10, 20, 40 V; Max precursor per cycle 5; precursor threshold Abs. threshold 200 counts; Rel. thresholf 0.01%; active exclusion: exclusion after 1 spectra, and released after 0.5 min. Hexakis (1H,1H,3H-perfluoropropoxy) phosphazene at m/z 922.0097, and purine at m/z 121.0508 were used for external mass calibration. The data were processed with Qualitative Analysis 10.0.

## Supplementary Information

Additional file 1: Figure S1. Hi-C heatmaps of Ac. Hap1 and Ac. Hap2. Figure S2. Hi-C interaction diagram of inversion. Figure S3. Inversion variation between chromosomes of different genomes. Figure S4. Comparison of HiFi reads to the inverted breakpoint of the structure. Figure S5. KEGG and GO enrichment analysis of the A. catechu expansion and contraction gene family. Figure S6. Dotplot between C. nucifera, E. guineensis, P. dactylifera and Ac. Hap1. Figure S7. Speculative evolutionary patterns of A. catechu chromosomes. Figure S8. Dotplot between AMK and Ananas comosus. Figure S9. The proportion of different types repeat sequence in four palm plants. Figure S10. Density distribution of LTR-RTs insertion time of P. dactylifera and Ac Hap2. Figure S11. Total length of gene regions in palm plants. Figure S12. The analysis of DEGs between different tissues of Ac. Hap1. Figure S13. KEGG enrichment analysis of DEGs. Figure S14. GO enrichment analysis of DEGs. Figure S15. Phylogenetic tree of MTs. Figure S16. Phylogenetic tree of UGTs. Figure S17. Phylogenetic tree of A.catechu MTs and other reported MTs. Figure S18. Mass spectrum of six standard. Figure S19. Mass spectrum of flavonoids. Figure S20. Distribution of specific chromosomal regions on haplotype-resolved genomes. Figure S21. Venn diagram showed the overlap between low expression genes (TPM<1) and SVs/TEs.

Additional file 2: Table S1. Summary of PacBio sequencing. Table S2. Summary of Hi-C sequencing. Table S3. The lengths of pseudochromosomes generated in the Hi-C assembly. Table S4. BUSCO analysis of genome and annotation. Table S5. Functional annotation of predicted protein-coding genes in the A. catechu genome. Table S6. Statistics of repeated elements in the A. catechu genome. Table S7. The identified centromeres in A. catechu of two haplotypes. Table S8. Statistics of structural variation and SNPs. Table S9. Average gene expression (TPM) of Ac. Hap1 in different tissues. Table S10. Average gene expression (TPM) of Ac. Hap2 in different tissues. Table S11. Species used for homology-based gene prediction and phylogenomic analysis. Table S12. Statistics of repeated elements of other species of Arecaceae. Table S13. Metabolic data from our previous study. Table S14. Differentially accumulated metabolites between three different tissues. Table S15. Differentially expressed genes (Ac. Hap1) between three different tissues. Table S16. Reported MTs used in this study. Table S17. All MTs and UGTs in A.catechu. Table S18. Correlation between DAMs and DEGs. Table S19. Primers used in this study.

## Data Availability

All data supporting the results of this study are included in the manuscript and its additional files. The whole genome sequence data reported in this paper have been deposited in the Genome Warehouse in National Genomics Data Center under BioProject accession number PRJCA033974. The RNA-seq presented in this study are available in NCBI under the BioProject accession PRJNA929020. The raw metabolomics data are available via http://www.ebi.ac.uk/metabolights/MTBLS6967.

## Abbreviations

MT: Methyltransferases
UGT: Uridine diphosphate glycosyltransferase
UDP-Glc: UDP-glucose
UDP-Rha: UDP-rhamnose
WGD: Whole genome duplication
AMK: Ancient monocot karyotypes
LTR: Long terminal repeat
SNP: Single nucleotide polymorphism
indels: Insertions or deletions
SyRI: Synteny and rearrangement identifier
Mya: Million years ago
NANMT: Nicotinate *N*-methyltransferase
GNMT: Guvacine *N*-methyltransferase
DEG: Differentially expressed gene
DAM: Differentially accumulated metabolites
