# Supplementary material for: Haplotype-resolved genome reveals haplotypic variation and the biosynthesis of medicinal ingredients in Areca catechu L
Source: Mol Hortic. 2025 May 2;5:24. doi: 10.1186/s43897-025-00146-2 (PMC12046898; doi:10.1186/s43897-025-00146-2)
Supplement: Supplementary file 1 — Additional file 1: Figure S1. Hi-C heatmaps of Ac. Hap1 and Ac. Hap2. Figure S2. Hi-C interaction diagram of inversion. Figure S3. Inversion variation between chromosomes of different genomes. Figure S4. Comparison of HiFi reads to the inverted breakpoint of the structure. Figure S5. KEGG and GO enrichment analysis of the A. catechu expansion and contraction gene family. Figure S6. Dotplot between C. nucifera, E. guineensis, P. dactylifera and Ac. Hap1. Figure S7. Speculative evolutionary patterns of A. catechu chromosomes. Figure S8. Dotplot between AMK and Ananas comosus. Figure S9. The proportion of different types repeat sequence in four palm plants. Figure S10. Density distribution of LTR-RTs insertion time of P. dactylifera and Ac Hap2. Figure S11. Total length of gene regions in palm plants. Figure S12. The analysis of DEGs between different tissues of Ac. Hap1. Figure S13. KEGG enrichment analysis of DEGs. Figure S14. GO enrichment analysis of DEGs. Figure S15. Phylogenetic tree of MTs. Figure S16. Phylogenetic tree of UGTs. Figure S17. Phylogenetic tree of A.catechu MTs and other reported MTs. Figure S18. Mass spectrum of six standard. Figure S19. Mass spectrum of flavonoids. Figure S20. Distribution of specific chromosomal regions on haplotype-resolved genomes. Figure S21. Venn diagram showed the overlap between low expression genes (TPM<1) and SVs/TEs. [file 43897_2025_146_MOESM1_ESM.docx]

**Supporting Information**

**Haplotype-resolved genome reveals haplotypic variation and** **the biosynthesis of medicinal ingredients in *Areca catechu* L.**

Chao Wang^1,2,†^, Lei Tan^3,4,†^, Zhonghui Zhang^1,2,†^, Xianggui Li^1,2,†^, Linghao Xia^1,2^, Peng Cao^1,2^, Haiyang Tong^1,2^, Xumin Ou^1,2^, Shixuan Li^1,2^, Jianing Zhang^1,2^, Chun Li^1,2^, Jun Yang^1,2,⁎^, Wen-Biao Jiao^3,4,⁎^ & Shouchuang Wang^1,2,⁎^

†Chao Wang, Lei Tan, Zhonghui Zhang, Xianggui Li contributed equally to this work.

*Correspondence:

Shouchuang Wang

shouchuang.wang@hainanu.edu.cn

Wen-Biao Jiao

jiao@mail.hzau.edu.cn

Jun Yang

yang9yj@hainanu.edu.cn

^1^ National Key Laboratory for Tropical Crop Breeding, School of Breeding and Multiplication (Sanya Institute of Breeding and Multiplication), Hainan University, Sanya Hainan 572025, China

^2^ National Key Laboratory for Tropical Crop Breeding, College of Tropical Agriculture and Forestry, Hainan University, Sanya Hainan 572025, China

^3^ National Key Laboratory for Germplasm Innovation & Utilization of Horticultural Crops, Huazhong Agricultural University, Wuhan, 430070, China

^4^ Hubei Hongshan Laboratory, Wuhan, 430070, China


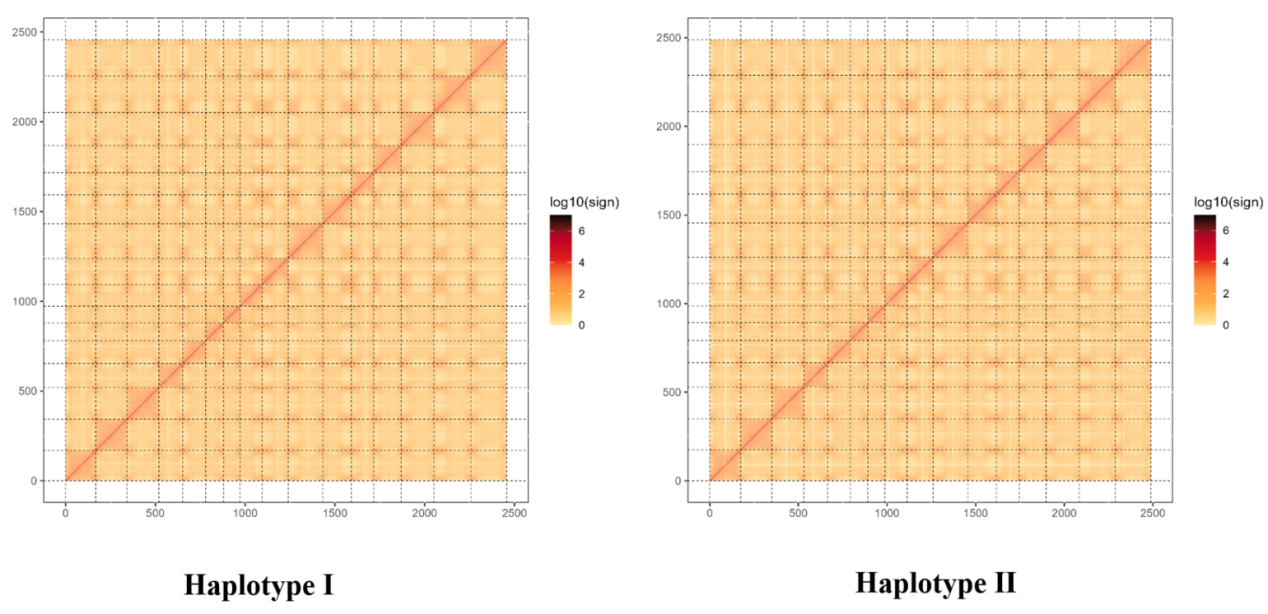


**Figure S1.** Hi-C heatmaps of *Ac. Hap1* and *Ac*. *Hap2.* The color from light yellow to dark red show low to high probability of interactions.

**Figure S2.** Hi-C interaction diagram of inversion. (A-C) the HiC interaction maps of three large inversion sites on chromosome 10, 12, and 13.

**
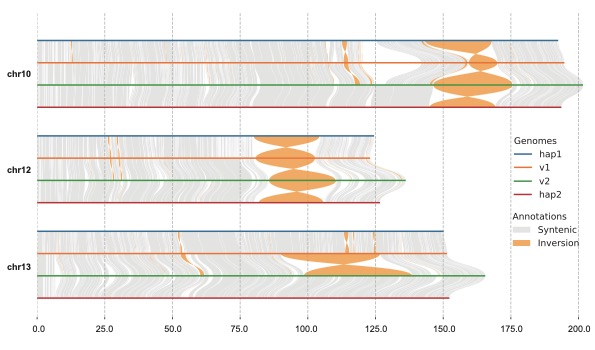
**

**Figure S3.** Inversion variation between chromosomes of different genomes. The collinearity between the two haplotypes assembled in this study and the previously published *A. catechu* genome on chromosomes 10, 12, and 13.

**Figure S4.** Comparison of HiFi reads to the inverted breakpoint of the structure. (A-C) represents the coverage of HiFi reads at three large inverted breakpoints on chromosome 10, 12, and 13, respectively.


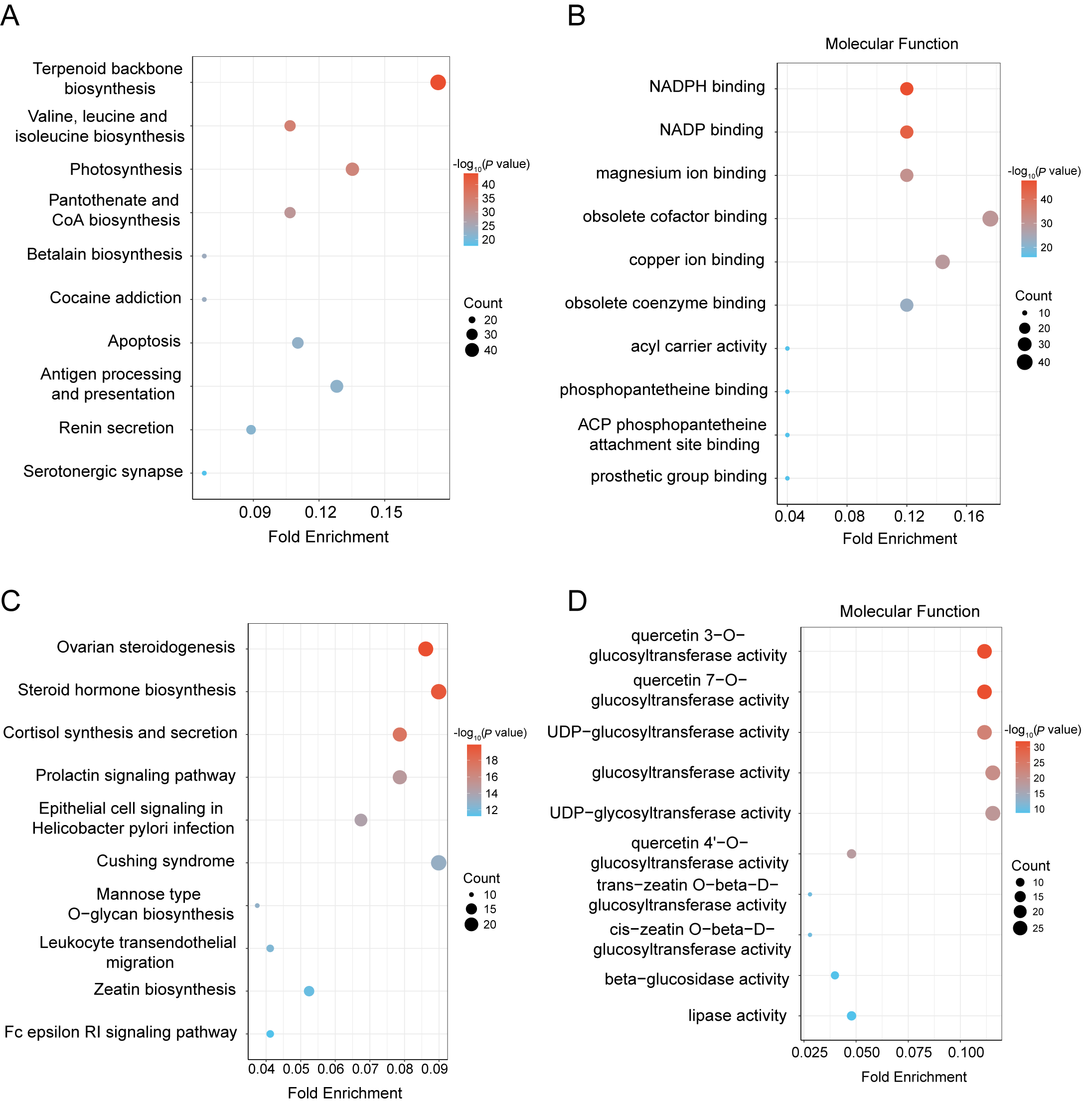


**Figure S5.** KEGG and GO enrichment analysis of the *A. catechu* expansion and contraction gene family. (A, B) KEGG (A) and GO (B) enrichment analysis of expansion gene family. (C, D) KEGG (C) and GO (D) enrichment analysis of contraction gene family.


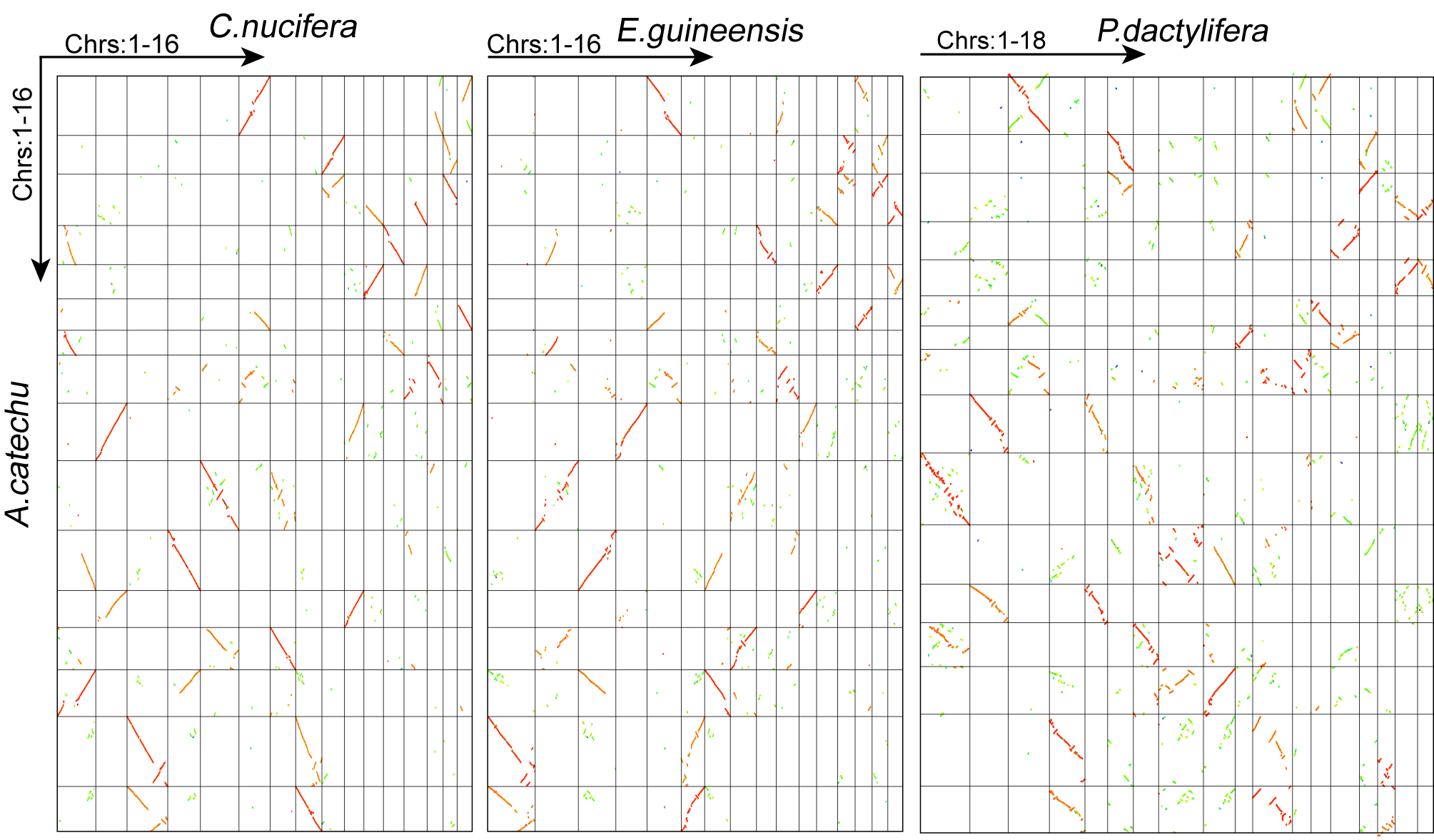


**Figure S6.** Dotplot between *C. nucifera*, *E. guineensis*, *P. dactylifera* and *Ac. Hap1*.


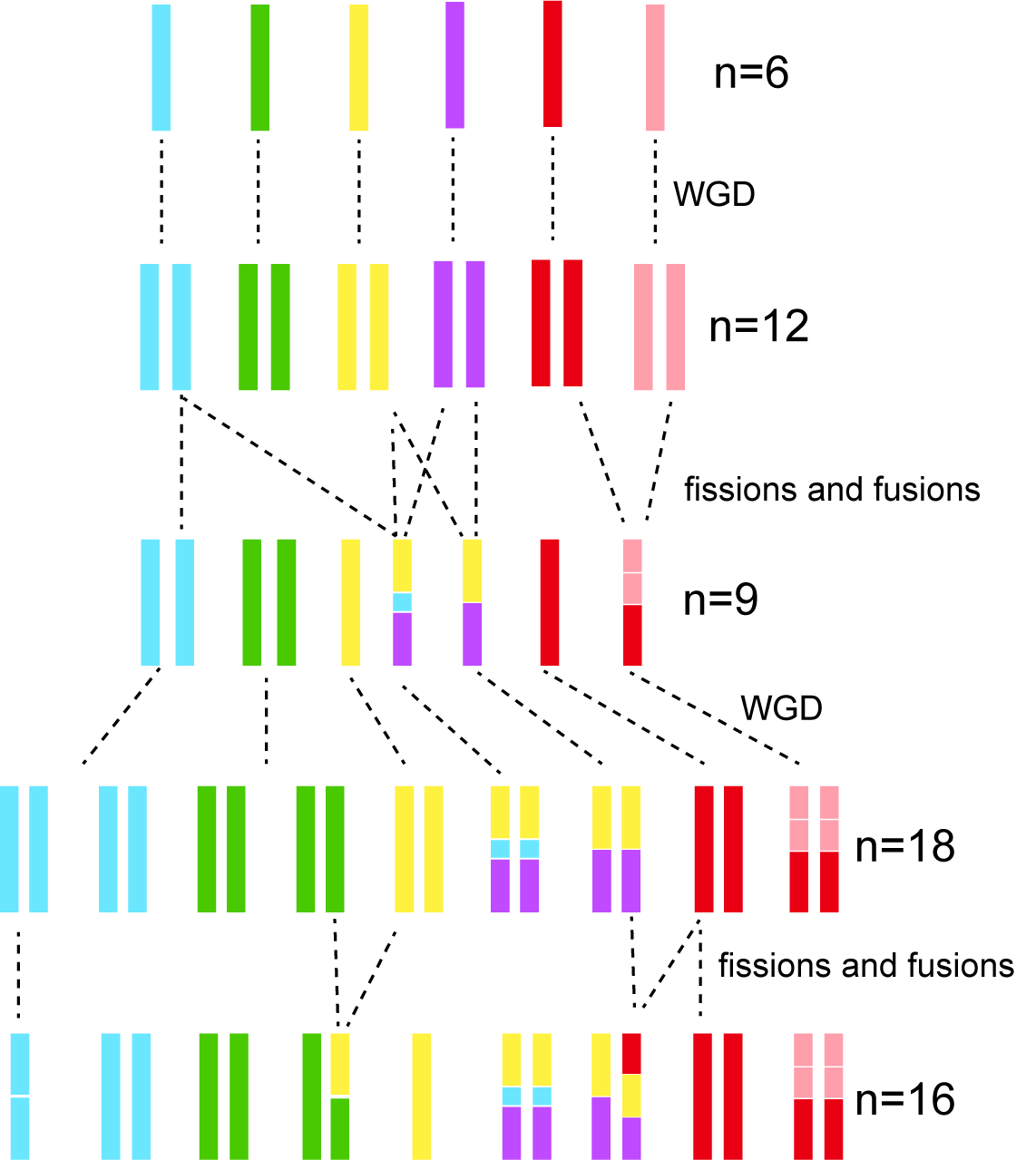


**Figure S7.** Speculative evolutionary patterns of *A. catechu* chromosomes.


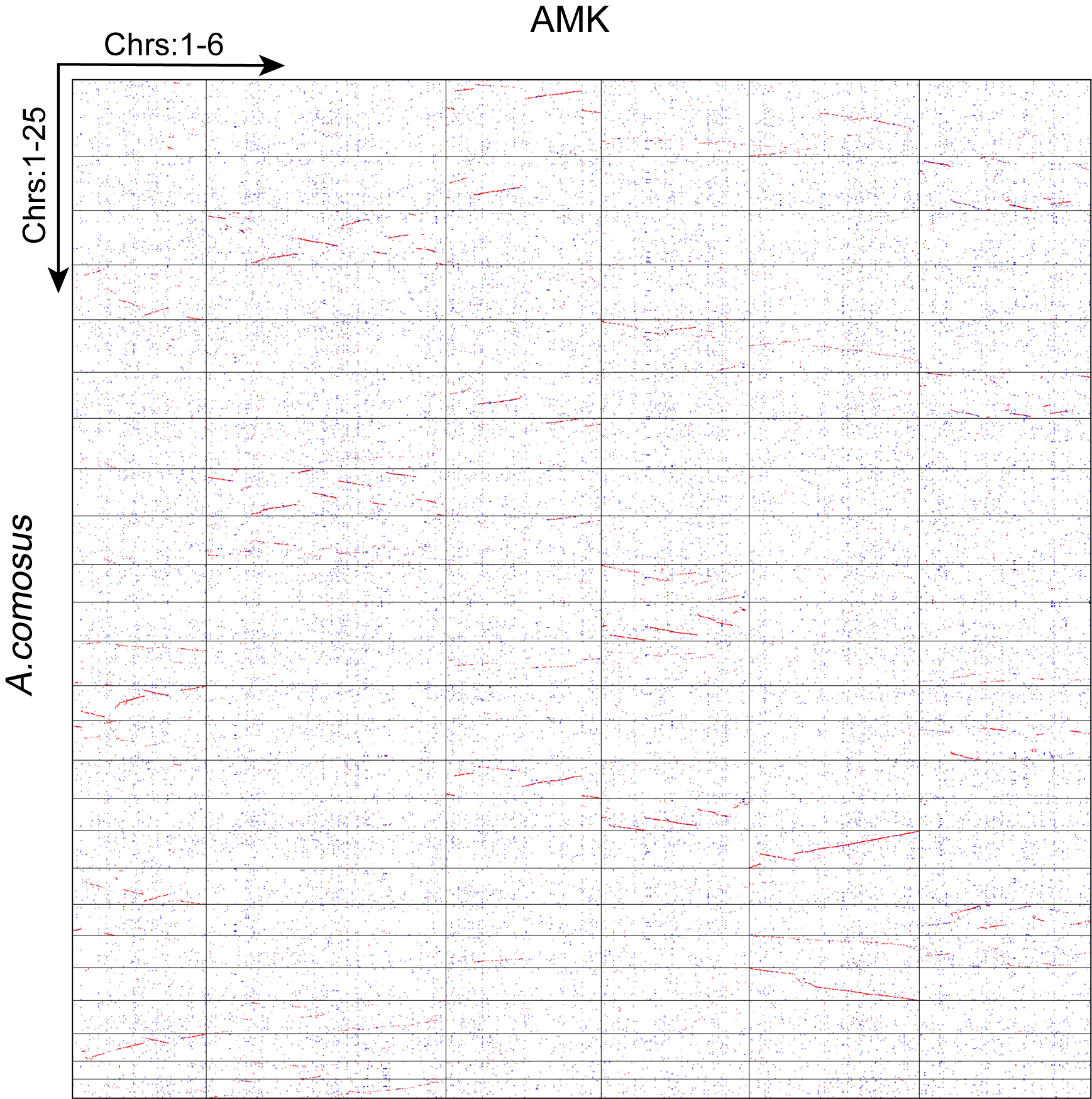


**Figure S8.** Dotplot between AMK and *Ananas comosus*.


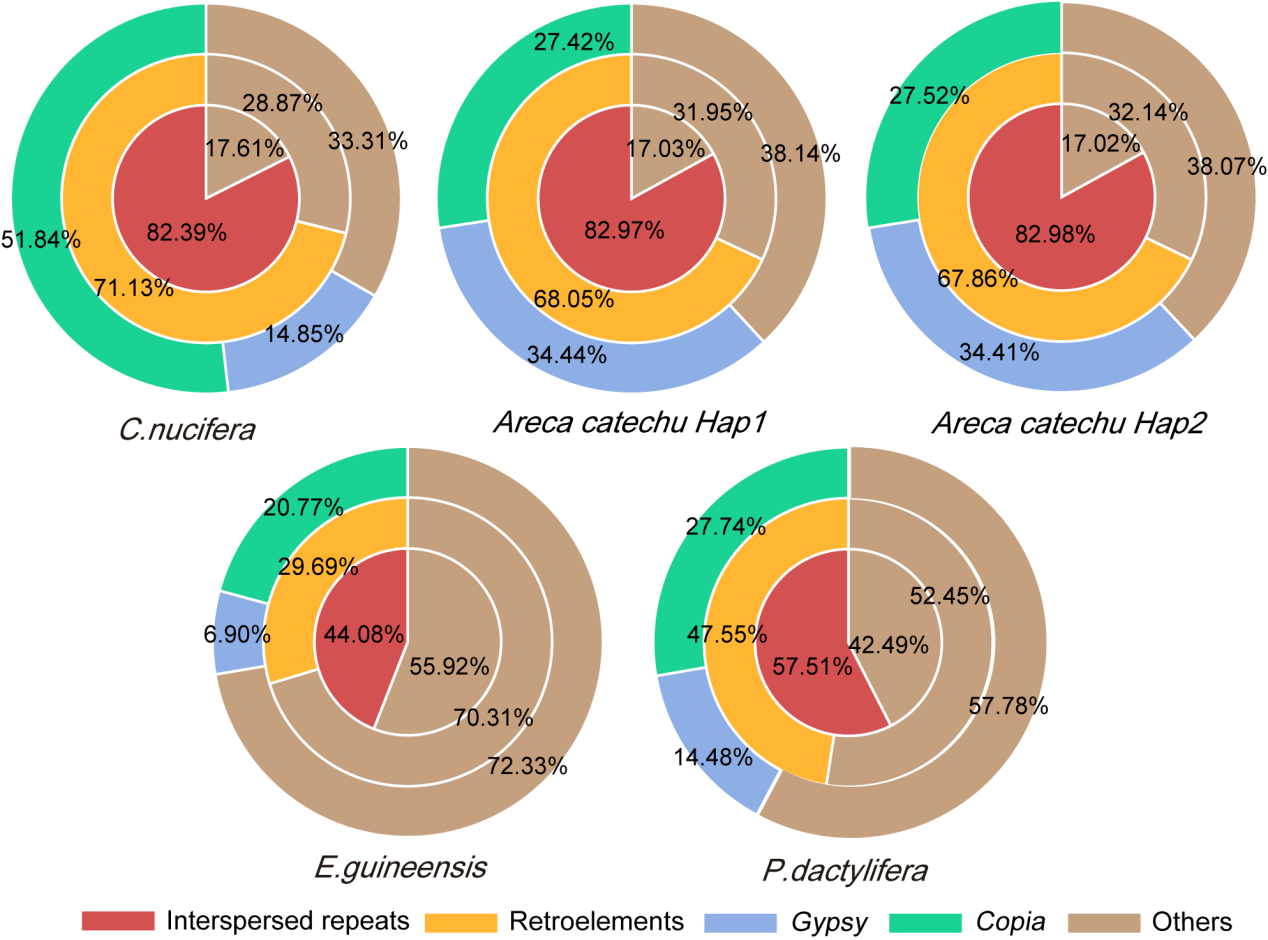


**Figure S9.** The proportion of different types repeat sequence in four palm plants.


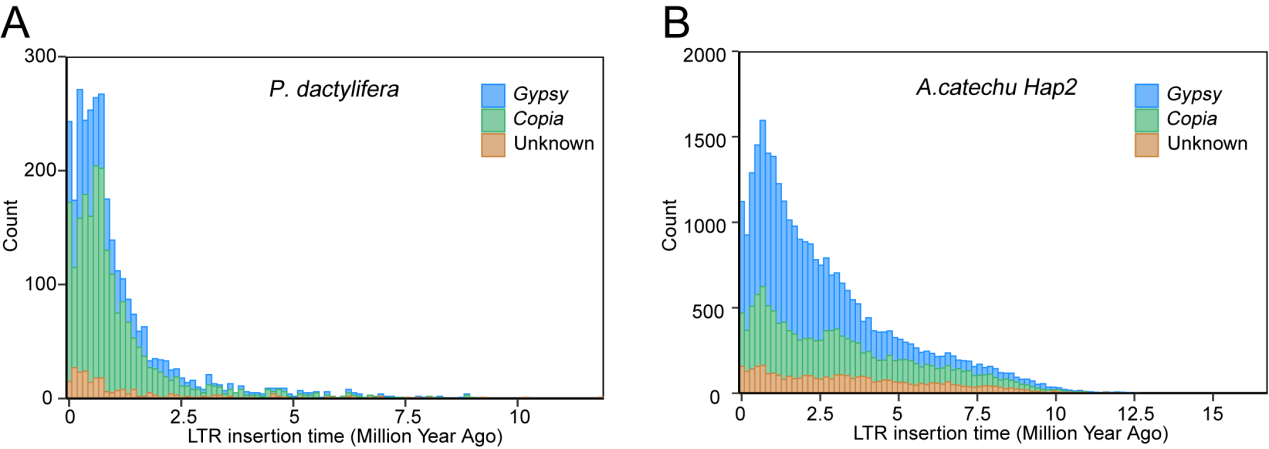


**Figure S10.** Density distribution of LTR-RTs insertion time of *P. dactylifera* and *Ac Hap2.*


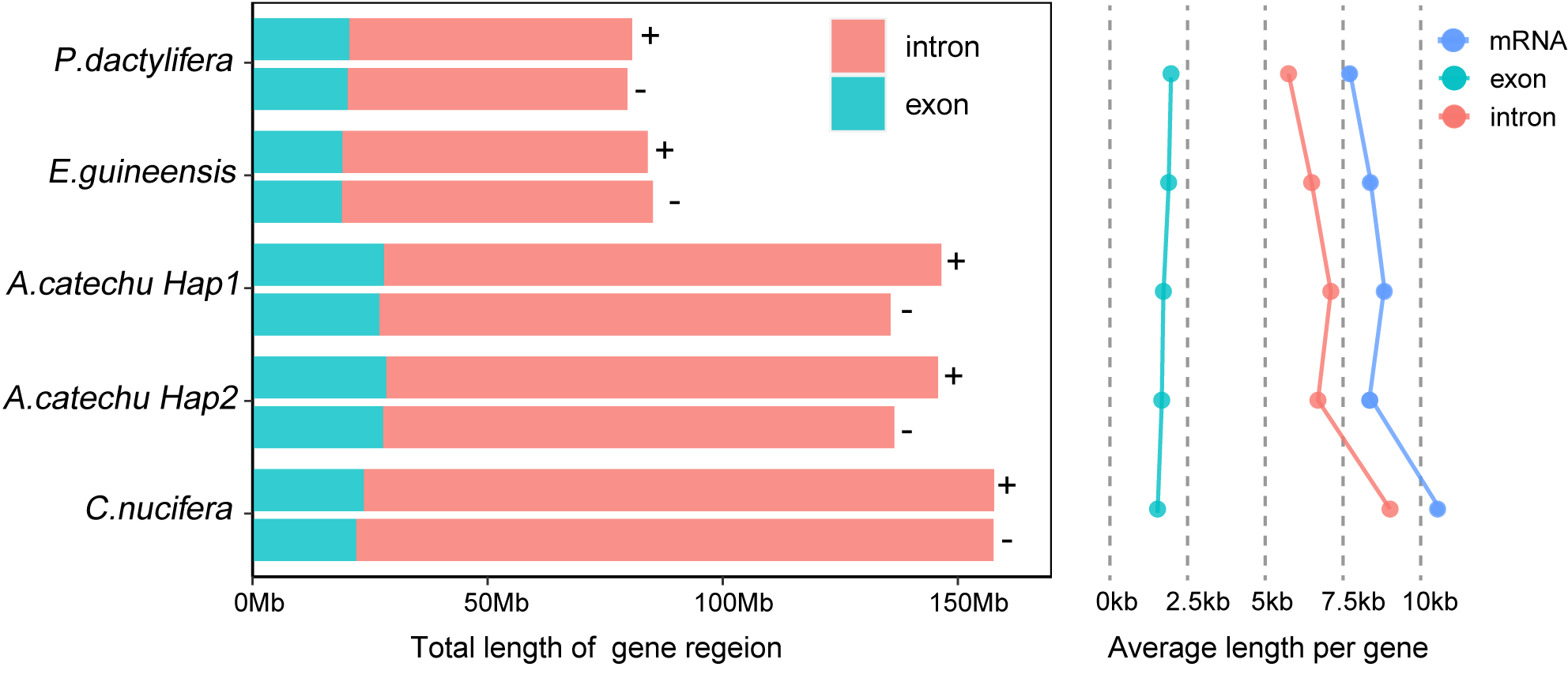


**Figure S11.** Total length of gene regions in palm plants.


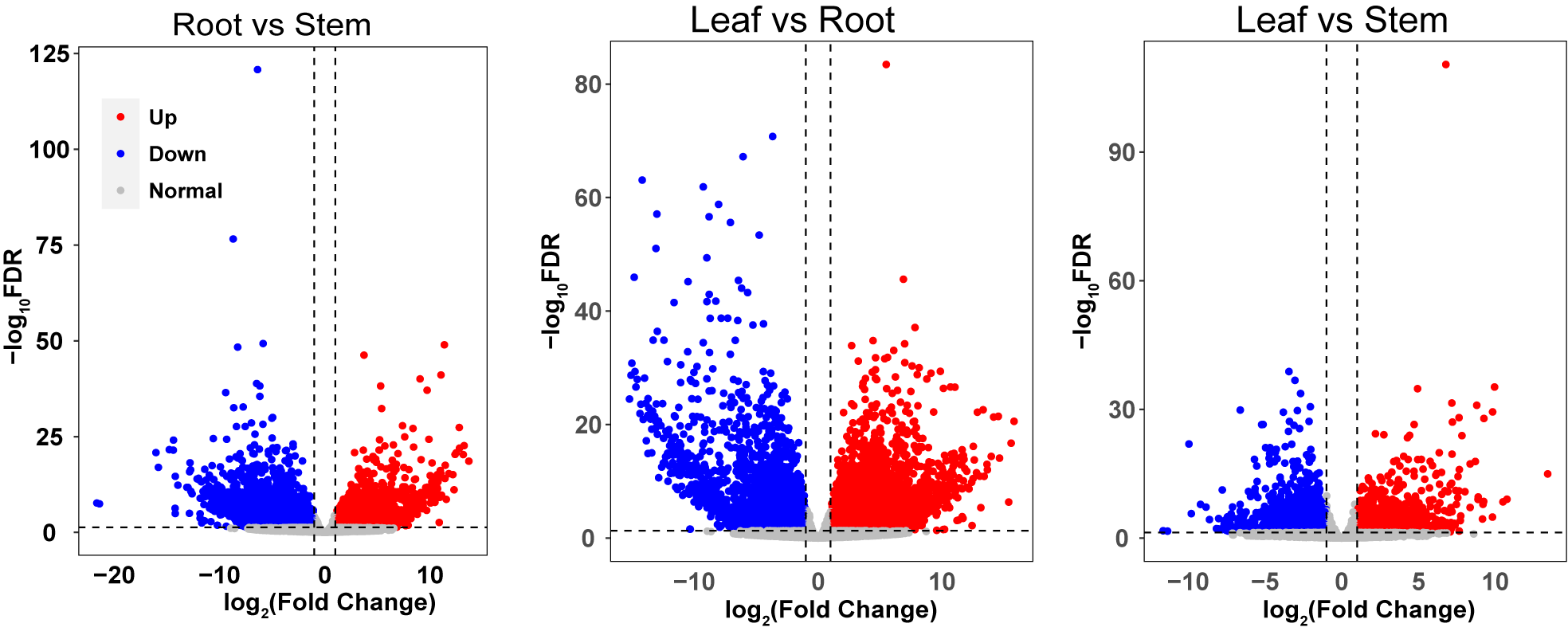


**Figure S12.** The analysis of DEGs between different tissues of *Ac. Hap1*. Blue dots represent downregulated genes, and red dots represent upregulated genes.


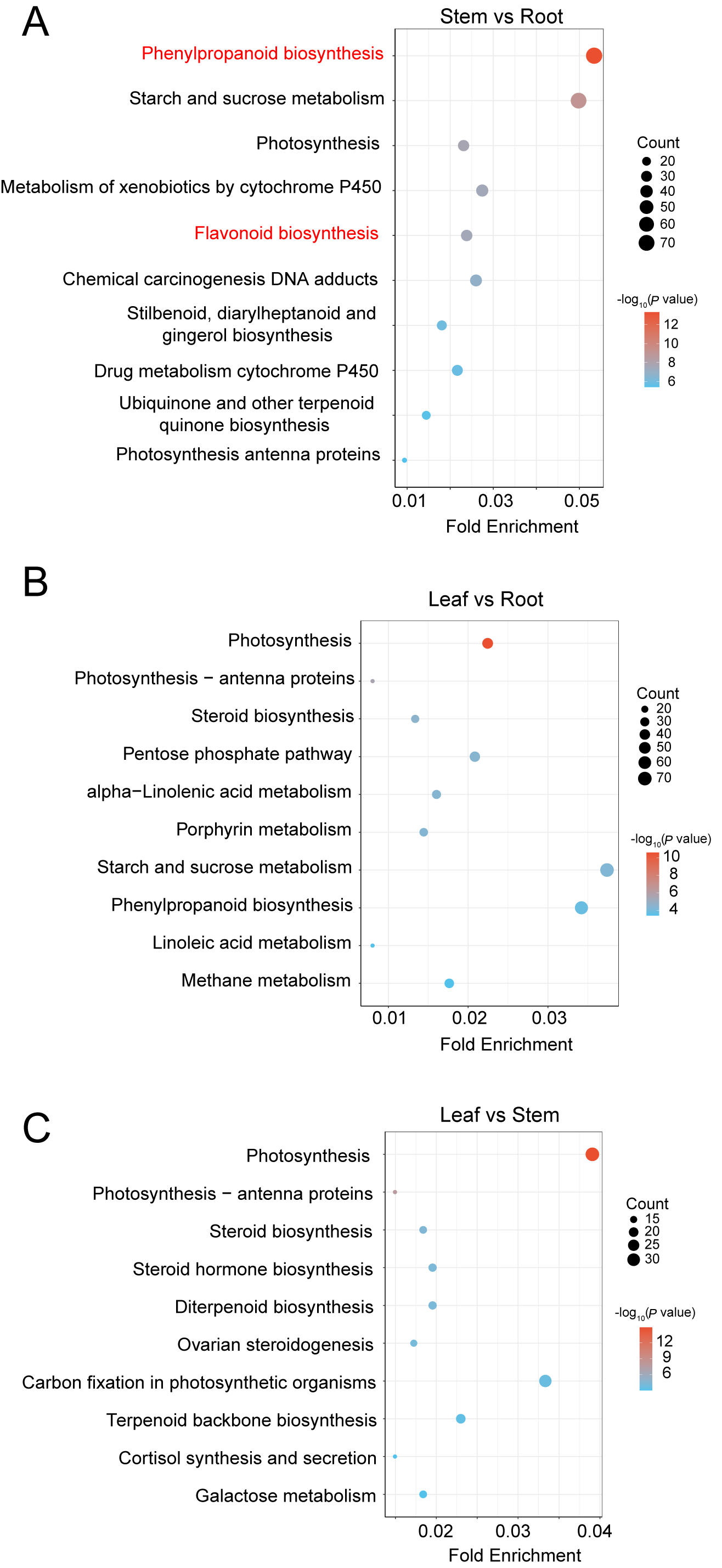


**Figure S13.** KEGG enrichment analysis of DEGs. (A-C) Bubble charts present KEGG enrichment of DEGs by comparing stem and root (A), leaf and root (B), and leaf and stem (C).


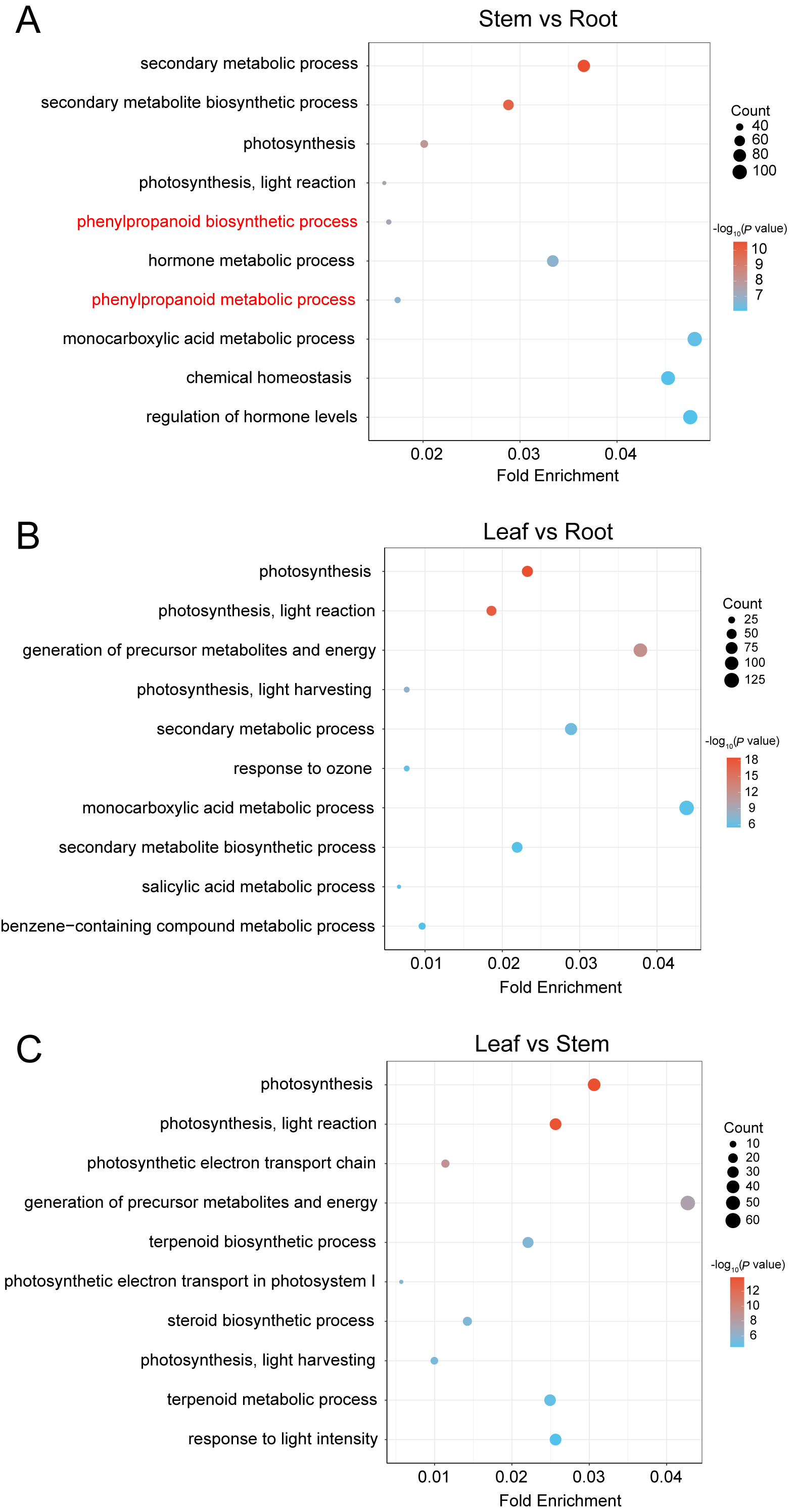


**Figure S14.** GO enrichment analysis of DEGs. (A-C) Bubble charts present biological process enrichment of DEGs by comparing stem and root (A), leaf and root (B), and leaf and stem (C).


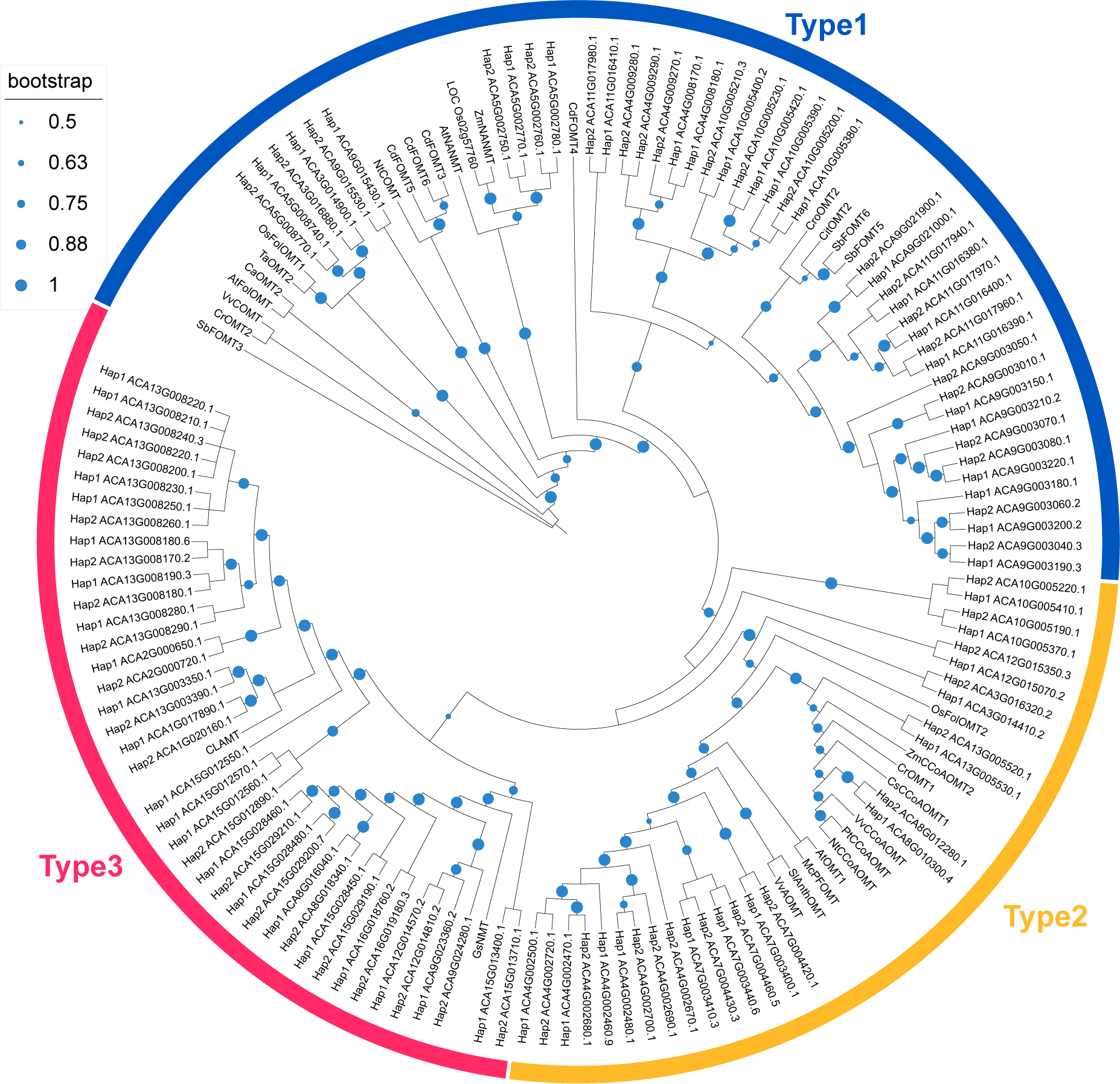


**Figure S15.** Phylogenetic tree of MTs. The increase in blue dots indicates an increase in bootstrap value from small to large. Type1 represent Caffeic acid-3-*O*-methyltransferase (COMT); Type2 represent Caffeoyl-CoA O-methyltransferase (CCoAOMT); Type3 represent others MT.


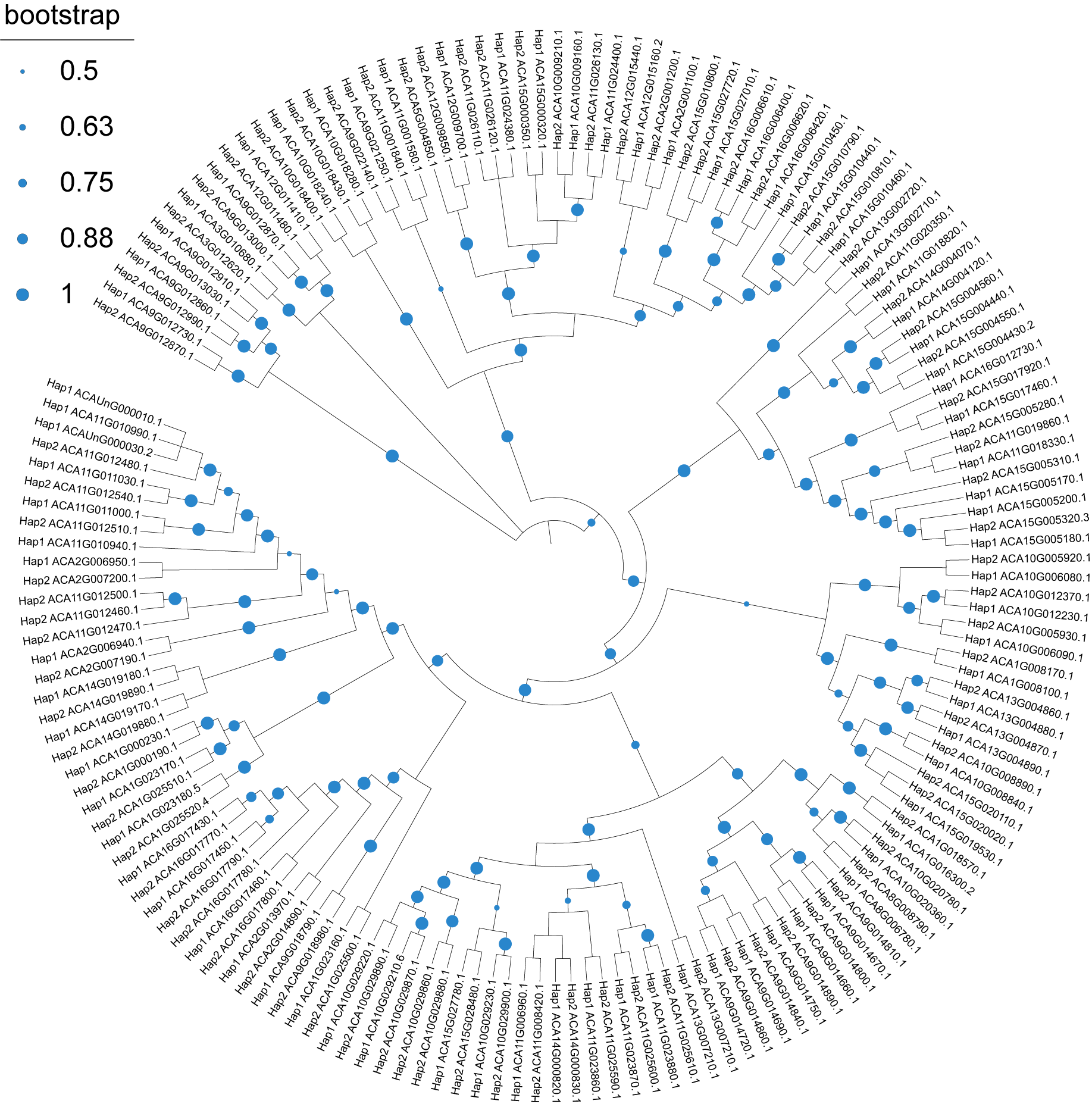


**Figure S16.** Phylogenetic tree of UGTs. The increase in blue dots indicates an increase in bootstrap value from small to large.


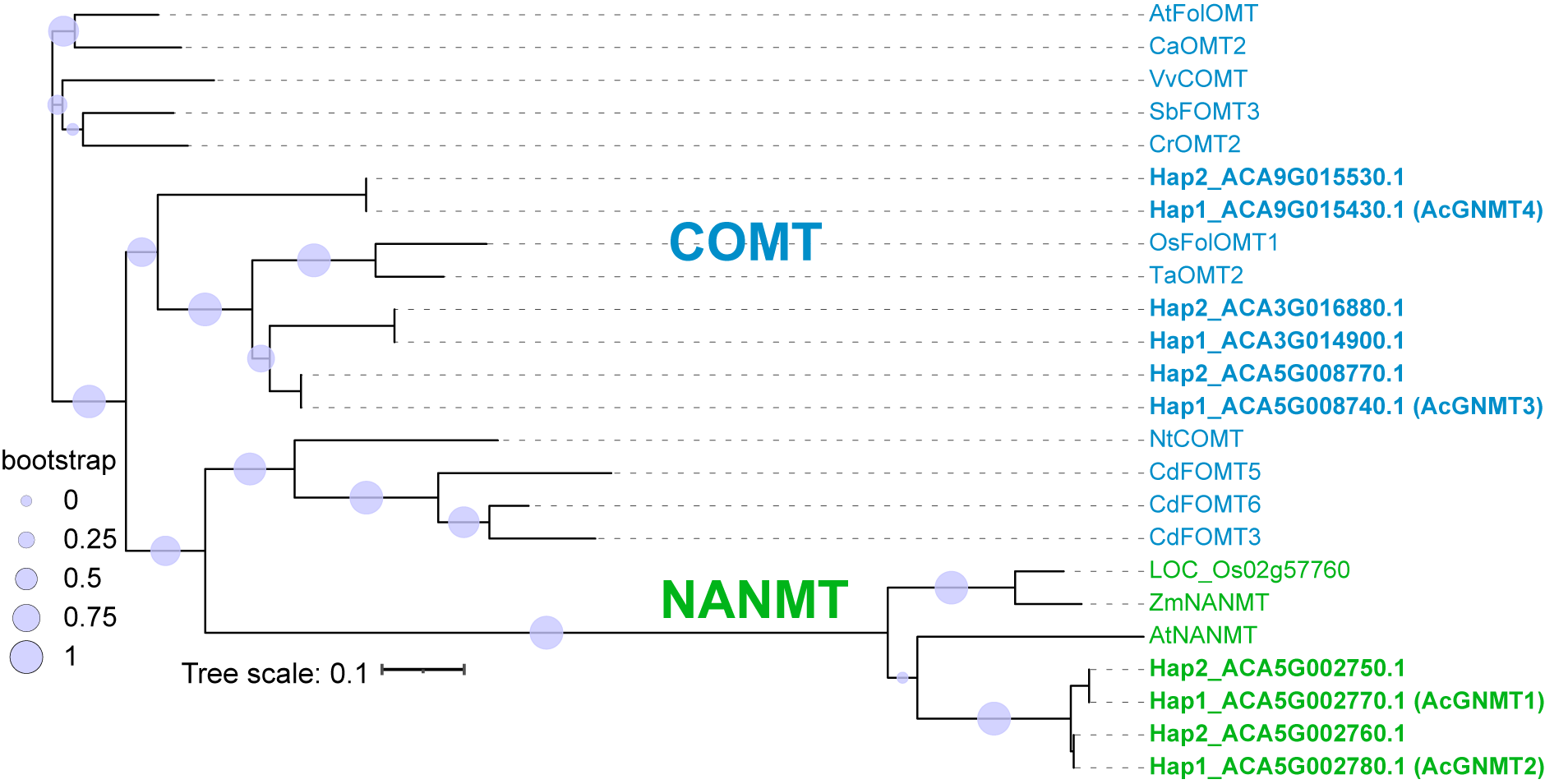


**Figure S17.** Phylogenetic tree of *A.catechu* MTs and other reported MTs. The increase in purple dots indicates an increase in bootstrap value from small to large. COMT, caffeic acid *O*-methyltransferase; NANMT, nicotinate *N*-methyltransferase.


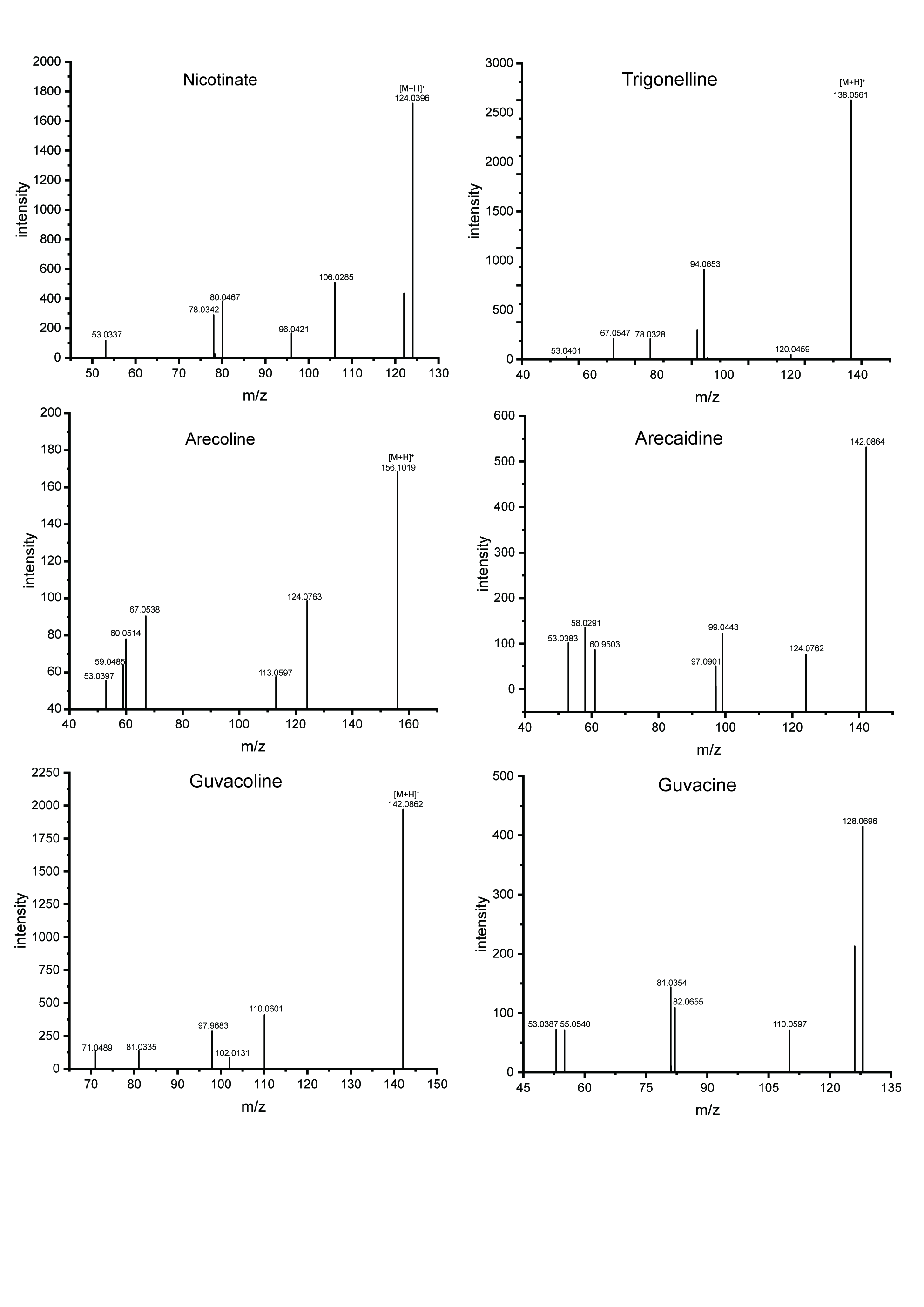


**Figure S18.** Mass spectrum of six standard (nicotinate, trigonelline, arecoline, arecaidine, guvacoline and guvacine).


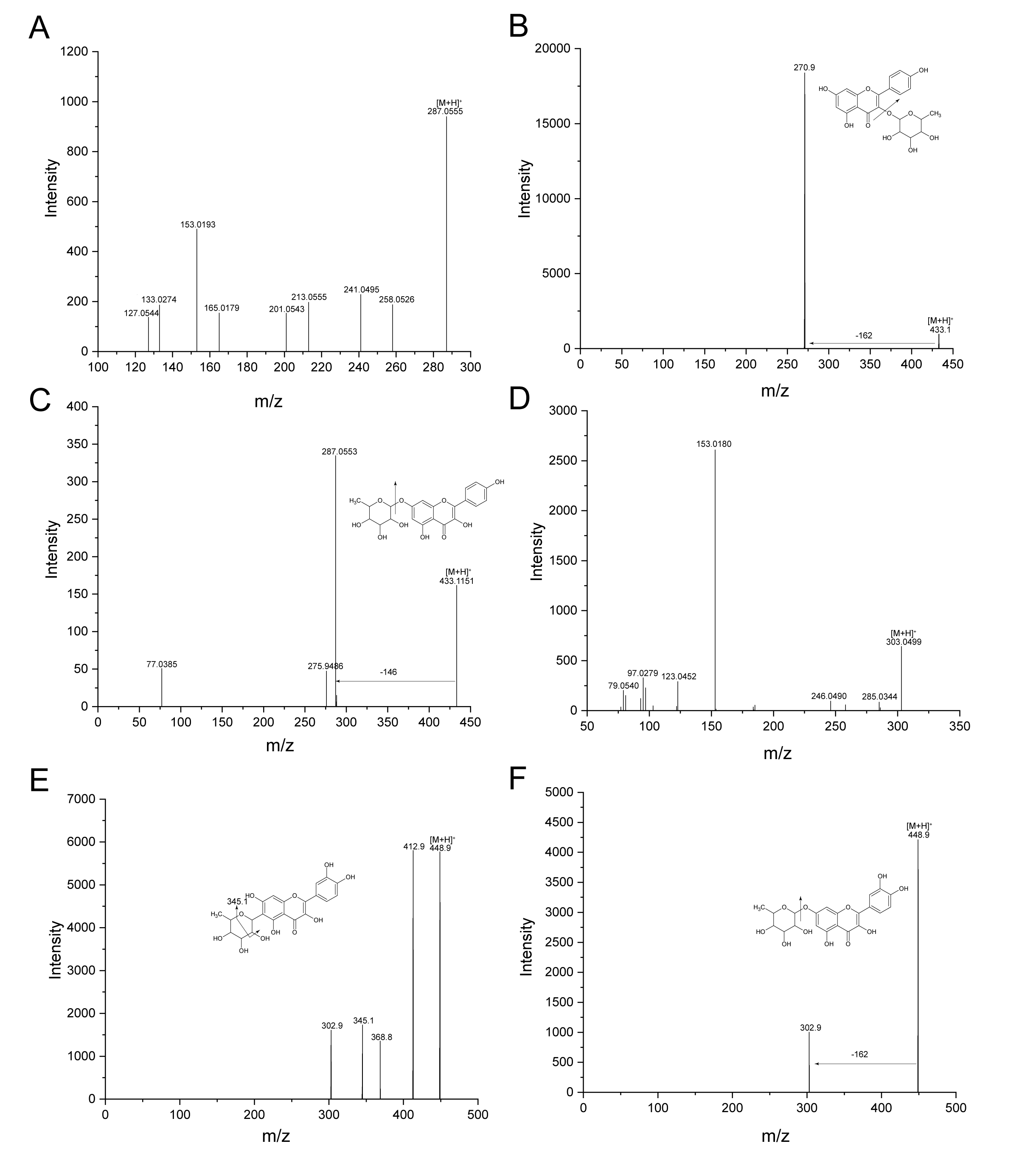


**Figure S19.** Mass spectrum of flavonoids. (A-C) MS/MS spectra of kaempferol (A) and kaempferol *O*-glucoside (B,C) (RT = 6.09 and 6.79 min). (D-F) MS/MS spectra of quercetin (D), quercetin *O*-glucoside (F, RT = 6.63 min) and quercetin *C*-glucoside (E, RT = 6.34 min).


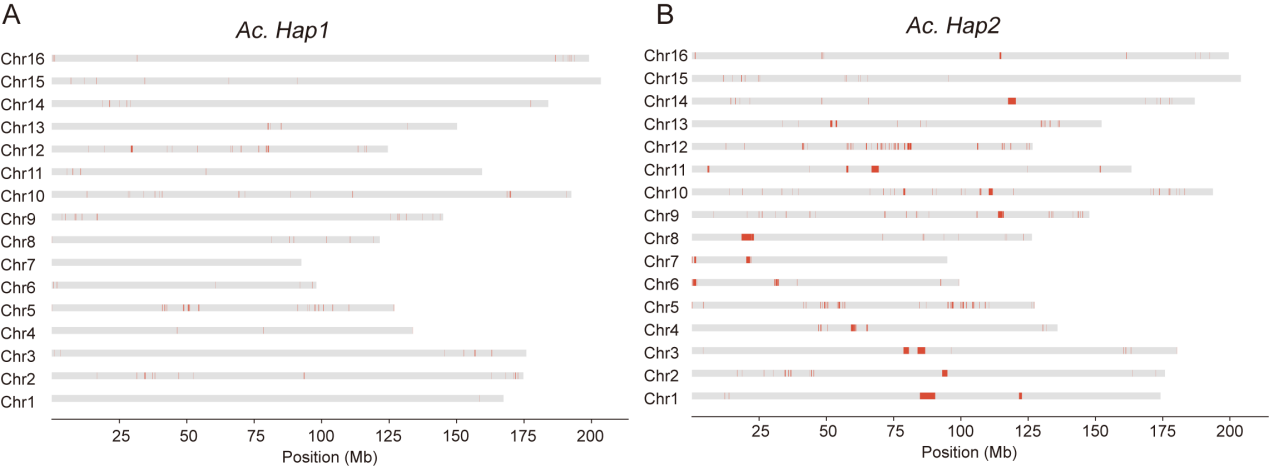


**Figure S20.** Distribution of specific chromosomal regions on haplotype-resolved genomes. Red regions represented specific chromosomal regions.


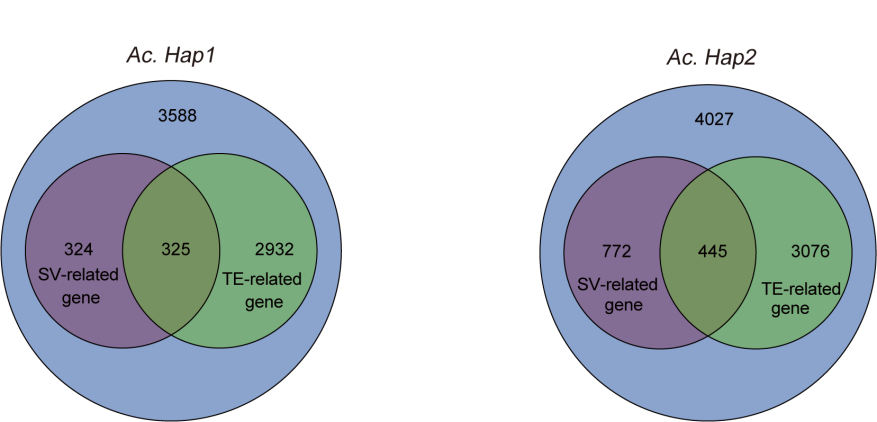


**Figure S21.** Venn diagram showed the overlap between low expression genes (TPM<1) and SVs/TEs. The blue circle represented the low expression genes, red circle represented that genes overlapped with SVs, and green represented that genes overlapped with TEs.
